# Adhesive cryogel particles for bridging confined and irregular tissue defects

**DOI:** 10.1186/s40779-023-00451-1

**Published:** 2023-03-23

**Authors:** Yao-Ting Xue, Ming-Yu Chen, Jia-Sheng Cao, Lei Wang, Jia-Hao Hu, Si-Yang Li, Ji-Liang Shen, Xin-Ge Li, Kai-Hang Zhang, Shu-Qiang Hao, Sarun Juengpanich, Si-Bo Cheng, Tuck-Whye Wong, Xu-Xu Yang, Tie-Feng Li, Xiu-Jun Cai, Wei Yang

**Affiliations:** 1 Department of Engineering Mechanics, Zhejiang University, Hangzhou, 310027 China; 2 Key Laboratory of Soft Machines and Smart Devices of Zhejiang Province, Zhejiang University, Hangzhou, 310027 China; 3 Center for X-Mechanics, Department of Engineering Mechanics, Zhejiang University, Hangzhou, 310027 China; 4 Department of General Surgery, Sir Run-Run Shaw Hospital, Zhejiang University, Hangzhou, 310016 China; 5 Soft Intelligent Materials Co., Ltd, Suzhou, 215123 China; 6 School of Biomedical Engineering and Health Sciences and Advanced Membrane Technology Research Centre, Universiti Teknologi Malaysia, 81310 Skudai, Malaysia

**Keywords:** Tissue reconstruction, Wet adhesion, Adhesive hydrogel, Bioadhesive

## Abstract

**Background:**

Reconstruction of damaged tissues requires both surface hemostasis and tissue bridging. Tissues with damage resulting from physical trauma or surgical treatments may have arbitrary surface topographies, making tissue bridging challenging.

**Methods:**

This study proposes a tissue adhesive in the form of adhesive cryogel particles (ACPs) made from chitosan, acrylic acid, 1-Ethyl-3-(3-dimethylaminopropyl) carbodiimide (EDC) and N-hydroxysuccinimide (NHS). The adhesion performance was examined by the 180-degree peel test to a collection of tissues including porcine heart, intestine, liver, muscle, and stomach. Cytotoxicity of ACPs was evaluated by cell proliferation of human normal liver cells (LO2) and human intestinal epithelial cells (Caco-2). The degree of inflammation and biodegradability were examined in dorsal subcutaneous rat models. The ability of ACPs to bridge irregular tissue defects was assessed using porcine heart, liver, and kidney as the ex vivo models. Furthermore, a model of repairing liver rupture in rats and an intestinal anastomosis in rabbits were established to verify the effectiveness, biocompatibility, and applicability in clinical surgery.

**Results:**

ACPs are applicable to confined and irregular tissue defects, such as deep herringbone grooves in the parenchyma organs and annular sections in the cavernous organs. ACPs formed tough adhesion between tissues [(670.9 ± 50.1) J/m^2^ for the heart, (607.6 ± 30.0) J/m^2^ for the intestine, (473.7 ± 37.0) J/m^2^ for the liver, (186.1 ± 13.3) J/m^2^ for muscle, and (579.3 ± 32.3) J/m^2^ for the stomach]. ACPs showed considerable cytocompatibility in vitro study, with a high level of cell viability for 3 d [(98.8 ± 1.2) % for LO2 and (98.3 ± 1.6) % for Caco-2]. It has comparable inflammation repair in a ruptured rat liver (*P* = 0.58 compared with suture closure), the same with intestinal anastomosis in rabbits (*P* = 0.40 compared with suture anastomosis). Additionally, ACPs-based intestinal anastomosis (less than 30 s) was remarkably faster than the conventional suturing process (more than 10 min). When ACPs degrade after surgery, the tissues heal across the adhesion interface.

**Conclusions:**

ACPs are promising as the adhesive for clinical operations and battlefield rescue, with the capability to bridge irregular tissue defects rapidly.

**Supplementary Information:**

The online version contains supplementary material available at 10.1186/s40779-023-00451-1.

## Background

In therapeutic practice, surgeons usually perform conventional suturing to reconstruct injured tissues, which are destructed automatically into fragments with confined and irregular defects. For instance, violent trauma can fracture limbs and organs, resulting in wounds with deep, narrow grooves [1–4]. The blood vessels and intestinal tracts may be severed during surgery, resulting in irregular annular cross-sections [5–8]. The reconstruction of tissues requires surface hemostasis and bridging separate tissues. However, bridging confined tissues with irregular surfaces is challenging. Suturing has been the most prevalent method for bridging tissues, but the procedure can be extremely time-consuming for irregularly shaped tissues [9] and has a high rate of leakage at the interfaces or through the pinholes [10, 11].

Adhesives are a promising way to bridge tissues [12]. Several tissue adhesives, including cyanoacrylate, fibrin, polyethylene glycol glues, nanoparticles, bioinspired adhesives, and hydrogels have been utilized. However, several drawbacks have been highlighted such as lack of biocompatibility (e.g., cyanoacrylate [13–15]) and weak adhesion to tissues (e.g., fibrin [16–18], polyethylene glycol [19, 20], nanoparticles [21], and bioinspired adhesives [22]). In contrast, adhesive hydrogels have excellent biocompatibility, and exhibit robust adhesion to tissues, controlled drug release, and wound management capabilities [23–26]. However, prefabricated hydrogels have limited applicability for confined and irregular tissue defects [27, 28]. Even though hydrogel tapes are able to adhere to the surfaces of tissues with ultra-strong and fault-tolerant adhesions [29], internal capillary leakage cannot be prevented as the tapes are applied outside the defects. For the case of hydrogel precursors applyed directly to arbitrary tissue defects, the result hydrogels are always weak, and the gelation process may require external stimuli (e.g., ultraviolet exposure [30], heating [31, 32], and pH change [27]), which are not applicable at tissue-tissue interfaces. Although pastes and dry particles based tissue adhesives possess advantages in applying to confined and irregular tissue defects [33–38], the undegradable agents along with those hydrogels would be retained in the tissues as obstacles to material exchange and tissue healing through the interfaces. A recent report showed a coacervate able to fit in irregular target sites [39], but it took a long time (approximately 10 min) to convert into a hydrogel and the lack of biodegradability limits its application between the tissue-tissue surface. In general, an ideal tissue adhesive must fulfill three requirements: 1) the adhesives must be able to adhere to the interfaces of confined and irregular tissue defects [6, 7]; 2) the interfacial adhesion must form rapidly and be strong enough to withstand imposed mechanical loads [40]; 3) the reserved adhesives should be biocompatible and biodegradable so that they do not impede material exchange and healing of tissues [12].

To bridge the confined and irregular tissue defects, we designed and synthesized the adhesive cryogel particles (ACPs), which were different from previous adhesives in preparation and application. In preparation, ACPs were synthesized through the process of freeze-drying and grinding a hydrogel. The resultant adhesives were particle like, distinct from most other adhesives such as tape, glue, and precursor in morphology. ACPs could be obtained via a rapid, mild, and cost-effective synthetic strategy, as the polyacrylic acid chains are linked to the chitosan chains through the 1-ethyl-3-(3-dimethylaminopropyl) carbodiimide (EDC)/N-hydroxysuccinimide (NHS) reaction, rather than using the expensive pretreated biological polymer. In application, ACPs differ from other adhesives by their ability to be applied not only to tissue surfaces but also to tissue-tissue interfaces. ACPs served as staples that bridge tissues point-by-point. The micro-sized ACPs can easily be applied to irregularly shaped tissue surfaces and form adhesions. The particles were fabricated to be porous, so that they could absorb residual water from tissues rapidly, becoming adhesive hydrogels without stimulation. The adhesive hydrogels contain functional groups forming instant and strong adhesion to tissue surfaces. Therefore, ACPs were utilized to bridge tissues within 10 s with interfacial adhesion energies comparable to the tissues' fracture energy. The biocompatibility and biodegradability in cell culture and dorsal subcutaneous implantation were also validated. This study demonstrates the application of ACPs on various destructed tissues in vivo and ex vivo is feasible. ACPs with adaptation to arbitrary tissue surfaces instantly exhibit strong adhesions and have excellent biocompatibility. They are promising as adhesives for bridging tissues in numerous clinical surgeries.

## Methods

### Ethics approval statement

All animal studies were approved by the Institutional Animal Care and Use Committee (IACUC) at Zhejiang University (#ZJU20220181) and postoperative care was supervised by the staff at the Animal Experimental Center of Sir Run-Run Shaw Hospital, Zhejiang University.

### Materials

To prepare the adhesive hydrogel, acrylic acid (AAc, Aladdin), chitosan (MW = 30,000, Macklin), α-ketoglutaric acid (α-keto, Sigma-Aldrich), NHS (Macklin), and EDC (Yuanye Bio-Technology) were used. Saline was used to purify the above-mentioned hydrogel and to remove the unreacted AAc. Liquid nitrogen was used to freeze-dry prepared hydrogels yielding ACPs. During the in vitro biodegradation test, Dulbecco’s phosphate-buffered saline (DPBS, without calcium and magnesium, Gibco), Lysozyme (Sigma-Aldrich), and Pancreatin (Solarbio) were used. In the 180-degree peel test, acrylamide (AAm, Aladdin), N, N’-Methylenebisacrylamide (MBAA, Sigma-Aldrich), and α-ketoglutaric acid (α-keto, Sigma-Aldrich) were used to prepare a tough hydrogel. In addition, the stiff backing layer applied for the tissues and tough hydrogel was made of poly (methyl methacrylate) films (70 μm in thickness, Anyuan Tech) and instant glue (PR100, 3 M).

### Hydrogel preparation

To prepare the adhesive, 0.02 g chitosan, 0.01 g EDC, and 0.004 g NHS were added to 5 ml deionized water, then stirred. 1 ml AAc was added dropwise into the mixture, and the chitosan dissolved completely. When the suspension liquid was transformed into a solution, 100 μl α-keto solution (0.1 mol/L in deionized water) was added to the precursor as an initiator. After ultrasonic defoaming, the prepared solution was transferred to a syringe and used as a mold under anaerobic conditions. The syringe was then exposed to ultraviolet (UV) radiation (365 nm, 500 mJ/cm^2^ power) for 60 min. The hydrogel was removed from the syringe and immersed in 250 ml saline solution for 72 h. The saline solutions were refreshed on daily basis to remove the residual acrylic monomer (Additional file 1: Fig. S1a, b).

### Preparation of ACPs

For the preparation of ACPs, the purified adhesive hydrogel block was immersed in liquid nitrogen and completely frozen. The frozen hydrogel was then ground for 30 s to obtain frozen hydrogel powder. The powder was freeze-dried for 72 h. ACPs were sealed in an aluminum foil bag and stored in a dry box with desiccants before usage (Additional file 1: Fig. S1c, d).

### Mechanical tests

Unless otherwise stated, all tissues and hydrogel samples were washed with PBS before dipping into ACPs, then pressed for 10 s (with 6.25 kPa pressure applied by weight). All the samples for mechanical tests were sliced into pieces of 2 cm in width and 8 cm in length. The thickness of the samples ranged between 2—5 mm depending on the geometric morphology of the tissues. A standard 180-degree peel test (5465, Instron) measured interfacial toughness at a constant peeling speed of 100 mm/min. On the representative force–displacement curve, the force increased and reached a plateau showing that the adhered sample is peeled in a steady state (Additional file 1: Fig. S2). The plateau force was calculated from the average value in the steady peeling process. The interfacial toughness equals twice the plateau force divided by the width of the sample. A poly(methylmethacrylate) film was attached to the samples using instant glue as the backing layer. The point acquired by the 180-degree peel test was repeated 3—5 times shown as mean and standard deviation.

### In vitro degradation test

To measure the degradability of ACPs, ACPs were immersed in two different enzyme solutions separately. For the degradation test in the pancreatin solution, ACPs (~ 0.1 g) were immersed in 10 ml of commercially available trypsin solution. For the degradation test in lysozyme solution, 500 μl of 1 mg/ml lysozyme aqueous solution was added to 10 ml DPBS. Then the pancreatin solution was replaced with lysozyme. All the samples were sealed in glass vials (20 ml) and incubated at 37 ℃ with shaking at 220 rpm. Every 2 d, the remaining ACPs were retrieved from the media. First, the sample was centrifuged to eliminate the degradation products of ACPs and salts from PBS. Then the ACPs were washed three times with deionized water. Finally, the sample was freeze-dried and weighed. The ratio of the mass of remaining lyophilized ACPs to that of the original ACPs was used to determine the degradation.

### Scanning electron microscopy (SEM)

ACPs and other tissues adhered to ACPs were probed with a scanning electron microscope (GEMINI 300, ZEISS). In detail, ACPs adhered tissue sample was cut into small square pieces (side width = 5 mm) followed by immersion in liquid nitrogen to fix the shape. Then, the cryo-frozen sample was lyophilized, and platinum sputtering was used to improve the image quality (Additional file 1: Fig. S3).

### Fourier transform infrared spectroscope (FTIR) characterization

A transmission FTIR (iS50, Thermo Fisher) with the KBr pellet method was used to characterize the composition of ACPs. Each spectrum was scanned 8 times with a wavenumber range of 4000—400 cm^−1^ and a resolution of 4 cm^−1^. The characteristic absorption peaks were marked in the FTIR spectrum (Additional file 1: Fig. S4).

### High-performance liquid chromatography (HPLC) characterization

Analytical HPLC with C_18_ column (HPLC; U3000, Thermo Fisher) was used to analyze the residual acrylic monomer from ACPs. The analyte for HPLC was prepared as follows. First, 10 ml adhesive hydrogel was incubated in 500 ml saline solution for 3 d with stirring. Unreacted AAc diffused from adhesive hydrogels to the saline solution until an equilibrium was reached. To avoid saturation, the saline solutions were refreshed daily. Then, the collected supernatant was filtered with a sterile 0.2 μm syringe filter and injected into the HPLC system for analysis. To obtain the calibration curve, a series of standard solutions of AAc with different concentrations i.e., 20 ng/ml, 50 ng/ml, 200 ng/ml, 300 ng/ml, and 500 ng/ml were used. In the HPLC procedure, the mobile phase is composed of two parts: A) 0.35% phosphoric acid aqueous solution (95% in volume percentage) and B) acetonitrile (5% in volume percentage). (Additional file 1: Fig. S5) The flow rate was fixed at 1 ml/min, the elution time was 10 min, and eluent detection was monitored at 210 nm. The concentration of the residual acrylic monomer in ACPs was calculated based on the calibration curve of varying acrylic acid monomer concentrations.

#### In vitro biocompatibility tests

In vitro biocompatibility tests were conducted using an ACPs-conditioned medium for cell culture (Additional file 1: Fig. S6). To prepare the ACPs-conditioned medium for in vitro biocompatibility tests, 1 mg of ACPs were incubated in 1 ml of Dulbecco’s modified Eagle medium (DMEM) supplemented with 10% fetal bovine serum, 1% penicillin, and 1% streptomycin at 37 ℃ for 24 h. Pristine DMEM without ACPs was used as a control. Either LO2 or Caco-2 cells were plated in 96-well plates at a density of 3,000 cells/well and maintained overnight in DMEM. Prepared cells were then treated with the ACP-conditioned media or pristine DMEM. After incubating at 37 ℃ for 24 h/48 h/72 h at 5% CO_2_, cell viability was evaluated by the LIVE/DEAD® Viability Assay Kit for mammalian cells (Thermo Fisher Scientific), which provides a two-color fluorescence cell viability assay that is based on the simultaneous determination of live and dead cells with two probes that measure recognized parameters of cell viability. The protocol was demonstrated below: 1) Thaw vials; 2) Transfer Live Green (Comp. A) into Dead Red (Comp. B); 3) Mix to create a 2X working solution; 4) Add an equal volume of 2X working solution to cells within 2 h; 5) Incubate for 15 min at 20 ℃—25 ℃; 6) Image cells. And a confocal microscope (LSM900, ZEISS) was used to image live cells (green) and dead cells (red) at excitation/emission wavelengths of 495 nm/515 nm and 495 nm/635 nm, respectively. Cell Counting Kit-8 (Yeason, China) was used to measure cell proliferation as indicated according to the manufacturer's instructions. A multiscan spectrophotometer (Thermo Scientific) was used to measure the absorbance at 450 nm.

#### In vivo biocompatibility and biodegradability tests

Female Sprague–Dawley rats (225 g to 250 g) were used for biocompatibility and biodegradability tests. All rats (*n* = 30) were randomly assigned to ACPs group (*n* = 15) and fibrin gel group (Hanbang, China) (*n* = 15) to evaluate the biocompatibility and biodegradability of ACPs. Subgroups as: ACPs D3 (*n* = 5), ACPs W1 (*n* = 5), ACPs W2 (*n* = 5), fibrin gel D3 (*n* = 5), fibrin gel W1 (*n* = 5), and fibrin gel W2 (*n* = 5). Before implantation, ACPs and the fibrin gel were prepared in a sterile environment. For implantation of ACPs or the fibrin gel in the dorsal subcutaneous space, rats were anesthetized using intraperitoneal administration of ketamine (80 mg/kg). The back hair of the rats was removed, and they were placed on a heating pad during the surgery. After a 1 cm skin incision in the center of the rat’s back was made, a 1/2 cm blunt dissection was performed from the incision toward the head of the rat to create a space for implantation. Either 1 mg of ACPs or 0.1 ml of fibrin gel were implanted subcutaneously. Up to three implants were placed per rat with the verification of absent overlap between each space. The back incision was closed by interrupted sutures (4–0 Prolene, Ethicon). All experiments were performed in a sterile environment. On day 3, week 1, and week 2 after implantation, the rats were euthanized by CO_2_ inhalation. Subcutaneous regions of interest were dissected and fixed in 10% formalin for 24 h for histological analyses. Blood was collected for analysis two weeks after the implantation (Additional file 1: Fig. S7).

#### In vivo liver rupture repair in rats

For liver rupture repair in rats, female Sprague–Dawley rats (225 g to 250 g) (*n* = 30) were randomly assigned to ACPs group (*n* = 15) and suture group (*n* = 15). Subgroups as: ACPs D3 (*n* = 5), ACPs W1 (*n* = 5), ACPs W2 (*n* = 5), suture D3 (*n* = 5), suture W1 (*n* = 5), and suture W2 (*n* = 5). The rats were anesthetized using intraperitoneal administration of ketamine (80 mg/kg). The abdominal hair was removed, and the rats were placed on a heating pad during the surgery. The liver was exposed via a midline abdominal incision. Surgeons used a biopsy punch (Miltex) to make an incision of 4 mm diameter and 7 mm depth into the liver. ACPs (2 mg) were applied in the punctured hole, and then gently pressed for 10 s to repair the rupture. For the suture group, interrupted sutures (5–0 Prolene, Ethicon) were also added to repair the rupture. Finally, the peritoneum was closed with continuous sutures (4–0 Prolene, Ethicon), and the abdomen was closed with interrupted sutures (4–0 Prolene, Ethicon). All experiments were performed in a sterile environment. On week 2 postoperatively, the rats were euthanized by CO_2_ inhalation and the blood was collected for analysis (Additional file 1: Fig. S8). Regions of interest from the liver rupture repair were dissected and fixed in 10% formalin for 24 h for histological analysis.

#### In vivo intestinal anastomosis of rabbits

For reconstruction of digestive tracts such as intestinal anastomosis, female New Zealand rabbits (2000 g to 2500 g) (*n* = 30) were randomly assigned to ACPs group (*n* = 15) and suture group (*n* = 15). Subgroups as: ACPs D3 (*n* = 5), ACPs W1 (*n* = 5), ACPs W2 (*n* = 5), suture D3 (*n* = 5), suture W1 (*n* = 5), and suture W2 (*n* = 5). After fasting for 24 h, the rabbits were anesthetized using intraperitoneal administration of ketamine (80 mg /kg). We removed the abdominal hair of the rabbits and placed them on a heating pad during the surgery. The small intestines were exposed via the midline abdominal incision. With its marginal vessels ligated, we performed side-to-side intestinal anastomosis with ACPs. The non-surgical area was covered by gauze to avoid unexpected contact with ACPs. ACPs were carefully spread on the side of the intestines with a small stainless picker. The intestine with ACPs was attached to another section of the intestine of the same length and held for 10 s.

Through a mini incision at the end of the anastomosis, we dissected the connective intestine 1 cm longitudinally and removed the intestine contents cleanly. After removing the intestinal contents, cleaning the lumen, and disinfecting the mini-incision, the mini-incision was closed by inverting interrupted sutures (6–0 Prolene, Ethicon) to achieve side-to-side intestinal anastomosis. A positive control group with conventional side-to-side intestinal anastomosis by incising the intestinal sides of 1 cm longitudinally and inverting interrupted sutures (6–0 Prolene, Ethicon) was set. All experiments were performed in a sterile environment. After 24 h, the rabbits were given a liquid diet, and a normal diet was given after 48 h. On week 2 postoperatively, blood was collected for analysis (Additional file 1: Fig. S9), and then the rabbits were euthanized by CO_2_ inhalation. Regions of interest of anastomosis sites were dissected and fixed in 10% formalin for 24 h for histological analyses.

#### Histological assessment

The blind histological assessment was conducted by a pathologist in the Department of Pathology, Sir Run-Run Shaw Hospital, Zhejiang University. The representative images were provided in the study. Histopathology of liver sections and intestine sections were examined with the scoring of inflammation (lymphocyte and neutrophil infiltration) [41]. The inflammation grade was demonstrated below: 1) Grade 0, no inflammatory cells; 2) Grade 1, < 10 inflammatory cells infiltration per high-power field (HPF); 3) Grade 2, > 10 inflammatory cells infiltration per HPF with ≤ 50% of the submucosa around the wound; 4) Grade 3, inflammatory cells infiltration with > 50% of the submucosa around the wound (Additional file 1: Fig. S10).

#### Statistical analysis

GraphPad Prism 8 (GraphPad Software, Inc., La Jolla, CA) was used to perform statistical significance of all comparison studies in this Article. In the statistical analysis for comparison between multiple samples, *Student’s t*-test was used, while a one-way analysis of variance (*ANOVA*) followed by *Tukey’s multiple comparison* test was conducted for comparison between multiple data groups. In the statistical analysis between two data groups, statistical significance and *P* values are determined by *Student’s t*-test. A *P* value of < 0.05 was considered significant.

## Results

### Mechanism of ACPs in bridging tissues

**Fig. 1:**
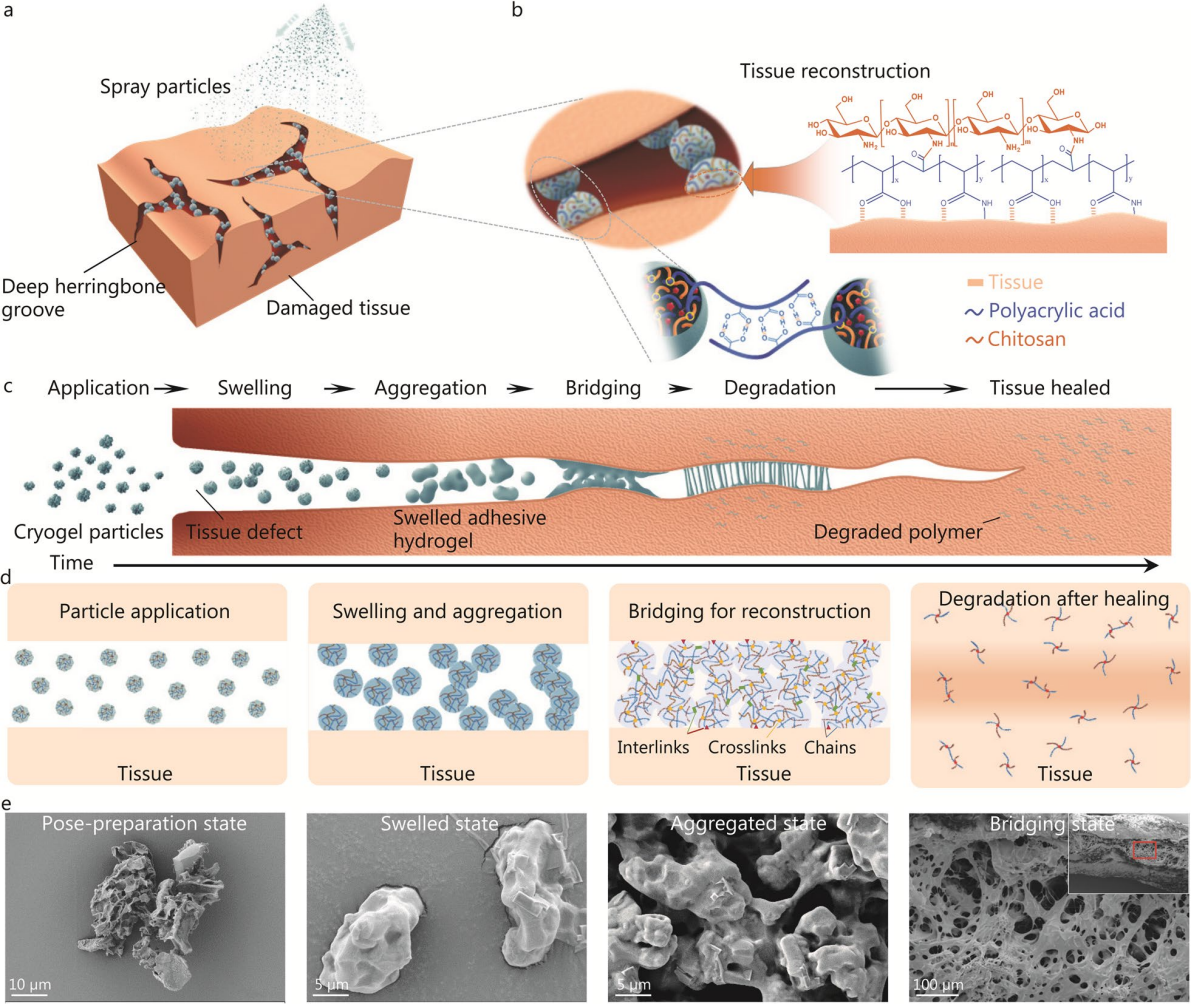
Schematics illustrating the mechanism of ACPs in bridging tissues. **a** ACPs are applicable at the interfaces between tissues for bridging. Deep herringbone grooves are representative confined and irregular topographies for severely injured tissues, and they are difficult for adhesives to bridge. **b** ACPs contain polymer networks of chitosan cross-linked with polyacrylic acid. They form hydrogel clusters at tissue interfaces, which bridge separate tissues point-by-point with hydrogen bonds and electrostatic interactions. **c** The morphology changes of ACPs and the evolution of tissue healing by applying ACPs. After being applied at tissue interfaces, ACPs swell by absorbing residual water, aggregate into adhesive hydrogel clusters, bridge tissues with bonds, and degrade when tissue heals. **d** The polymer chain evolution and the bond formation of ACPs while applying, swelling/aggregating, bridging tissues, and degrading. **e** Scanning electron microscope images of ACPs in the pose-preparation state, swelled state, aggregated state, and bridging state. The bridging state is captured after ACPs are used in bridging two pieces of the porcine intestine. ACPs adhesive cryogel particles

ACPs are designed to bridge tissues in four stages as follows (Fig. 1). 1) Application: ACPs are ground to micron size so they can be applied to and fit in micron-scaled tissue defects to conform to confined and irregular surface topographies. ACPs are small but resistant to being blown away, and they can be applied to tissue surfaces via dusting, dipping, etc. (Fig. 1a). 2) Aggregation: The tissue surfaces are often moist and bloody. ACPs are designed to be porous to rapidly absorb remaining tissue moisture. After absorption, the ACPs swell into hydrogel particles with sticky properties. The hydrogels consist of chitosan-crosslinked polyacrylic acid polymer networks that facilitate hydrogen bonding between the particles (Fig. 1b). As a result, ACPs swell and aggregate into adhesive hydrogel clusters. 3) Bridging: The polymer network of polyacrylic acid has rich carboxylic acid groups. They can rapidly form hydrogen bonds and electrostatic interactions with tissue surfaces. By adhering securely to both sides of the interface of tissue defects, hydrogel clusters connect these tissues. 4) Degradation: Chitosan, the crosslinking agent for hydrogel polymer networks, degrades when the glycoside linkages on its backbone are cleaved by enzymes. As chitosan degrades, the crosslinker density of the polymer network decreases, and the hydrogel changes from an insoluble solid to a polymer solution. With tissue renewal, degradation ensues. The reconstructed tissue gradually heals, and ACPs are eliminated. The chemical and physical changes of ACPs applied between tissues over time could be divided into the abovementioned 6 states i.e., application, swelling, aggregation, bridging, degradation, and tissue healing (Fig. 1c). Further, we picked 4 typical stages (particle application, swelling and aggregation, bridging for reconstruction, and degradation after healing) to illustrate the changes inner ACPs (Fig. 1d).

To create this design, micro-sized, porous, adhesive, biocompatible, and biodegradable ACPs were manufactured. Hydrogels composed of chitosan cross-linked polyacrylic acid networks were synthesized (Additional file 1: Fig. S11). Chitosan has rich amide groups, and it forms a peptide linkage by condensation with carboxylic acid groups on acrylic acid. EDC together with N-hydroxysuccinimide (NHS) was added to accelerate the condensation. A chitosan chain performs as the biodegradable crosslinker of the polymer network after it is condensed with more than one acrylic acid monomer. The condensation was validated by the presence of C-N–C units, which could be detected by using the transmission FTIR spectrum (Additional file 1: Fig. S4). The hydrogel was cured by free radical polymerization of acrylic acid under UV light. The hydrogel was then purified with saline solution to remove the toxic residual acrylic acid monomer from polyacrylic acid, which is biocompatible (Additional file 1: Fig. S5). The resulting hydrogel was adhesive, soft (elastic modulus of 0.46 kPa), and highly flexible (capable of stretching to 59.3 folds) (Additional file 1: Fig. S12). The adhesive hydrogel was freeze-dried into cryogel and ground into particles (Additional file 1: Fig. S1). The resulting ACPs had diameters of ~ 10 μm and were porous (pore size ~ 1 μm). During the adhesion process, the morphology of ACPs under through a series of transformations in the swelling, aggregating, and bridging procedure. After application, the porous ACPs absorb moisture and swell, ACPs quickly transform into non-porous hydrogel particles. Then, these hydrogel particles aggregate with each other and turn into a bulk hydrogel with channels. The bulk hydrogel adheres to the tissue surfaces, and forms bridging at tissue-tissue interfaces (Fig. 1e). The mechanical properties of the bulk hydrogel were related to the water content (Additional file 1: Fig. S13).

### Adhesion performance

The adhesion techniques of ACPs can be summed up as point-by-point bonding, with many points forming surfaces with arbitrary topographies. This adhesion strategy has been widely discovered in nature (e.g., starfish stick on rough reefs using their numerous tube feet and weaver ants weave leaves to form nests using silk) and has inspired the integration of soft materials by tough bridging islands, named molecular staples. In this strategy, the adhesion is robust once the diameters of the bridging points are smaller than the flaw sensitivity length of the adhesive.

**Fig. 2:**
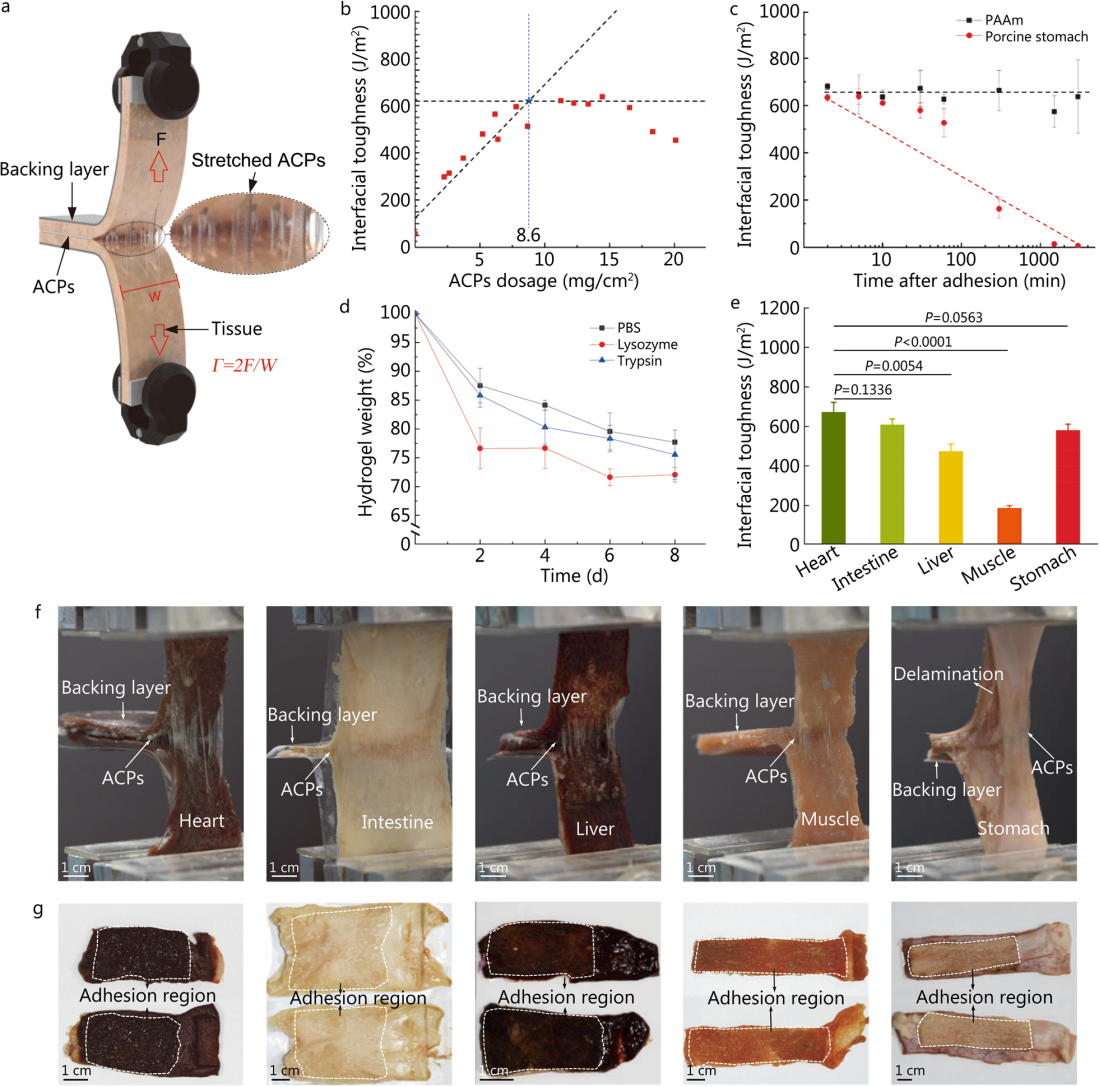
Interfacial adhesion performance of the ACPs. **a** 180-degree peeling setup for interfacial adhesion energy test. **b** Adhesion energy for hydrogels bridged by different doses of ACPs. The guidelines were plotted by linear fitting. **c** Adhesion energies as functions of time for hydrogels (ACPs do not degrade) and stomach pieces (ACPs degrade) bridged by the ACPs. **d** In vitro biodegradation of ACPs in PBS, PBS with lysozyme and pancreatin solution. **e** Adhesion energy of various tissues bridged by ACPs. *P* values are determined by *Student’s t*-test. **f** During the 180-degree peeling tests, ACPs elongate and bridge the tissues before the interfacial crack growth. **g** After peeling off, ACPs remain on both sides of the tissues, indicating that a cohesive failure has gone through ACPs layers. Values in **c**-**e** represent mean ± SD (error bars indicate SD; *n* = 3–5 independent samples). ACPs adhesive cryogel particles, PBS phosphate-buffered saline

A 180-degree peel test was used to evaluate the adhesion performance of ACPs. In this test, ACPs were applied between two pieces of the same material for adhesion. The adhesion energy, *Γ*, was calculated by dividing the two-fold measured peeling force, *F*, with the width of the specimen, *W* (Fig. 2a). First, ACPs dosage was assessed. A polyacrylamide (PAAm) hydrogel is selected as the model material for the measurement since it is highly accessible with stable physical characteristics that resemble soft tissues. Figure 2b shows that the adhesion energy drastically increases with low ACPs dosage (less than 8.6 mg/cm^2^), indicating the amount of ACPs was inadequate to cover the surface. When ACPs dosage exceeded 8.6 mg/cm^2^, adhesion energy barely changed and was constant at around 612.9 J/m^2^. The effect of degradation on adhesion performance was assessed. PAAm hydrogels were prepared with deionized water, which cannot promote degradation. As a result, ACPs remained stable at the PAAm interfaces, and the adhesion energy was maintained after ACPs were applied for 3000 min. In contrast, secretion from a porcine stomach and porcine intestine may contain various enzymes to degrade ACPs. The adhesion energy of those tissues by ACPs declines to zero after 1500 min (Fig. 2c). We also detected the weight loss of ACPs after they degraded and dissolved in the presence of lysozyme or trypsin enzymes (Fig. 2d). The enzymes in the stomach and intestine are capable of rapidly degrading ACPs. Whereas the adhesion energy measured ex vivo only accounts for the adhesion by ACPs, the tissues heal fast in vivo. The degradation rate should match the healing rate of the tissues so that ACPs will not retard tissue healing.

ACPs can bridge various tissues, including the porcine heart, intestine, liver, muscle, and stomach. Tissue toughness and its capacity to form bonds with ACPs change the adhesion performance. 180-degree peel test was applied to each tissue 3—5 times with independent samples to acquire the mean and the standard deviation. Consequently, the interfacial adhesion energy varies for different tissues [(670.9 ± 50.1) J/m^2^ for the heart, (607.6 ± 30.0) J/m^2^ for the intestine, (473.7 ± 37.0) J/m^2^ for the liver, (186.1 ± 13.3) J/m^2^ for muscle, and (579.3 ± 32.3) J/m^2^ for the stomach] (Fig. 2e). ACPs swell and aggregate into adhesive hydrogel clusters at the tissue interfaces. When the interfaces are opened by peeling, the hydrogel clusters are highly elongated before rupture. This is especially true in the case of hearts, which have the highest adhesion energy since the hydrogel clusters are enormously stretched upon peeling (Fig. 2f). The connection between the elongation of the hydrogel cluster and the adhesion energy is attributed to the toughening mechanism of crack bridging, and the deformation of the hydrogel cluster dissipates the energy at the crack tip. The interactions between ACPs and tissue were robust. Cohesive failure demonstrates that interfacial adhesion strength is larger than aggregated hydrogel strength (Fig. 2g).

### Biological performance

**Fig. 3:**
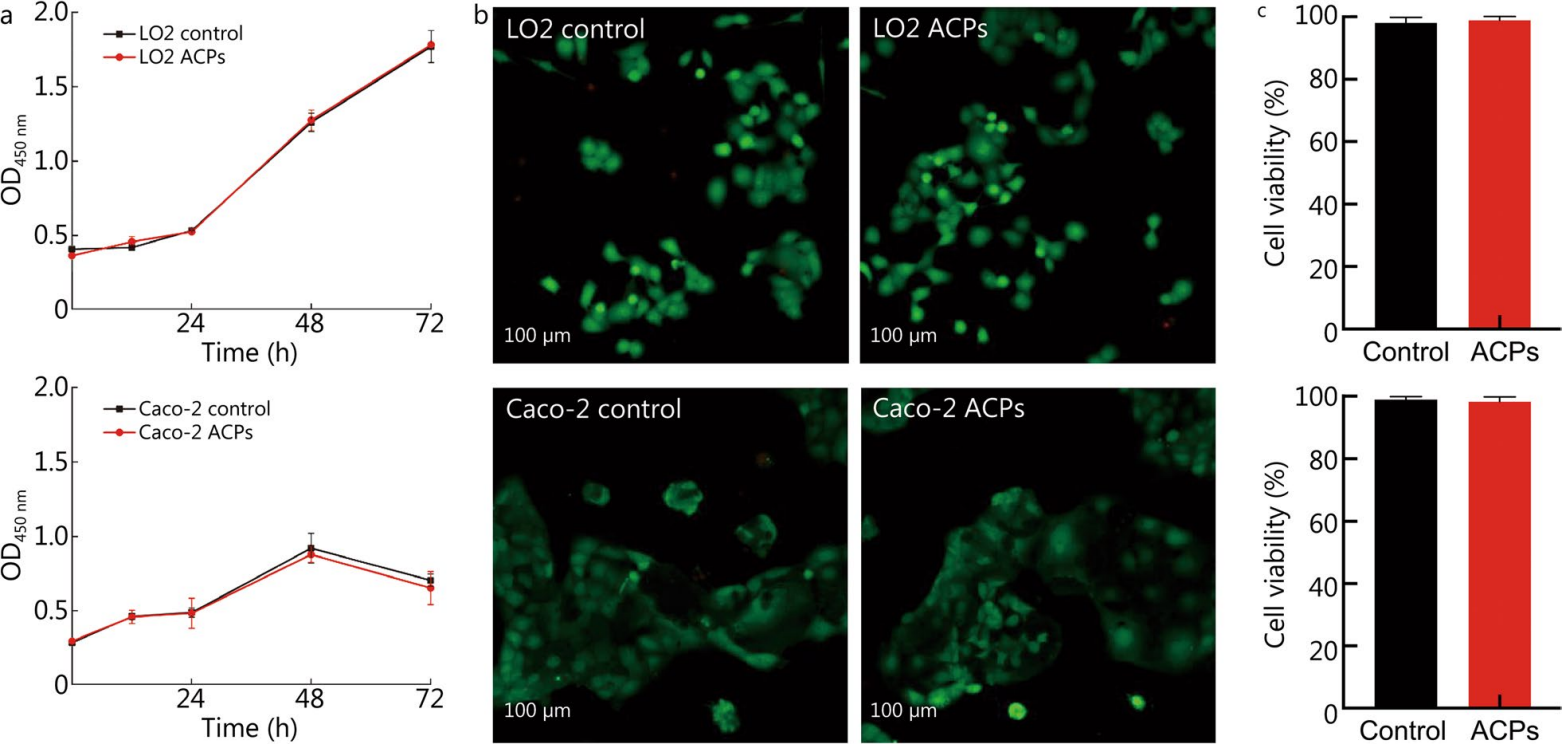
Biocompatibility of ACPs in vitro. **a** Fluorescent absorbance of LO2 and Caco-2 when they are cultured in a control medium and ACPs-conditioned medium. **b** Confocal microscopy images of the live/dead assay of LO2 (upper) and Caco-2 (lower) in control and ACPs-conditioned medium for 3 d.** c** In vitro cell viability of LO2 (upper) and Caco-2 (lower) in a live/dead assay after 3-day culture. ACPs adhesive cryogel particles, LO2 human normal liver cells, Caco-2 human intestinal epithelial cells

**Fig. 4:**
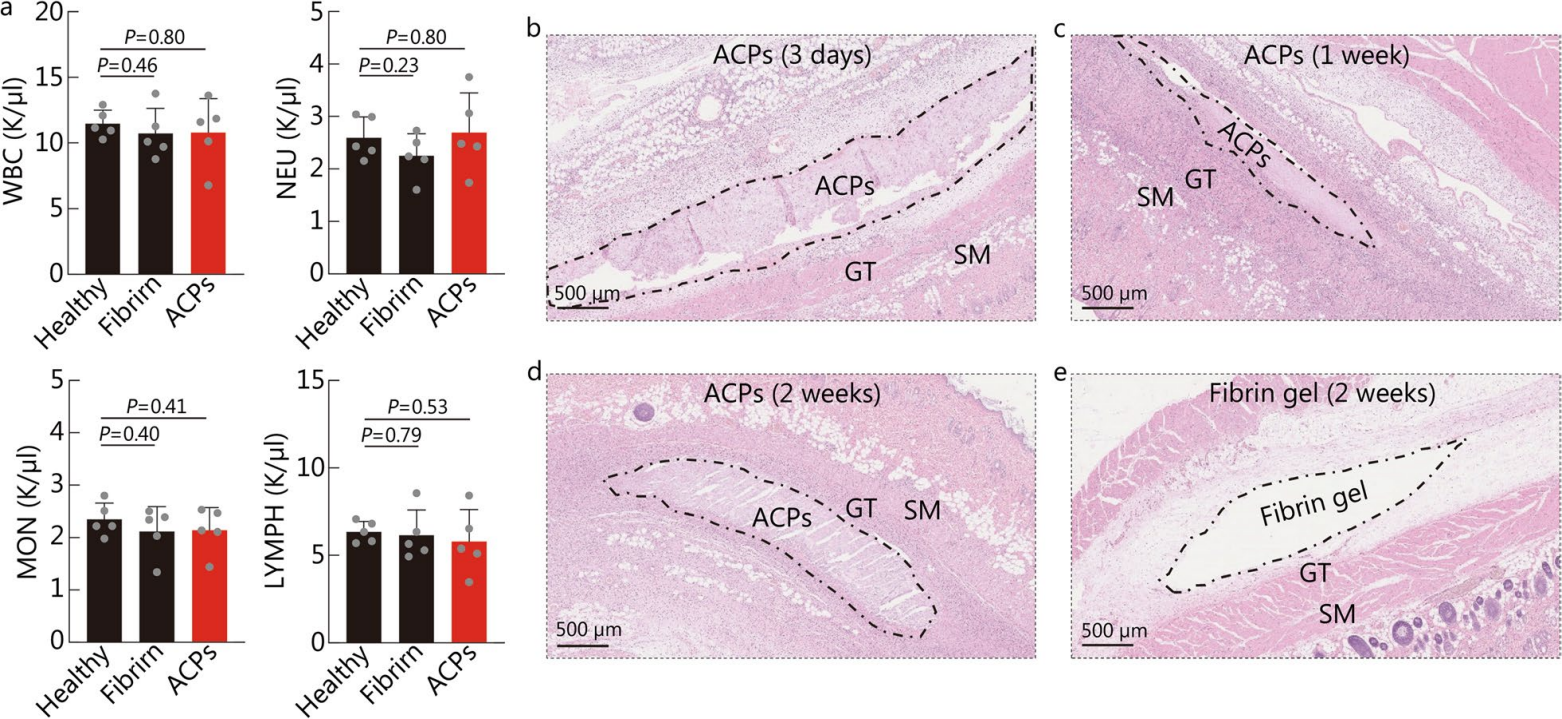
Biocompatibility of ACPs in vivo. **a** Representative blood analysis data of rats after ACPs implant for 2 weeks. **b**-**e** Representative histological images of ACPs after they are planted in the dorsal subcutaneous space for 3 d (**b**), 1 week (**c**), and 2 weeks (**d**). **e** Representative histological images of the fibrin gels' position after they are planted in the dorsal subcutaneous space for 2 weeks (fibrin gels would fall off during the H&E staining process). For each image, similar results are obtained in 4 additional independent experiments. ACPs adhesive cryogel particles, GT granulation tissue (indicate the areas of inflammation), SM skeletal muscle

We examined the biocompatibility of ACPs by cell culture in vitro and dorsal subcutaneous implantation in vivo. The cell proliferation of LO2 and Caco-2 was measured (Fig. 3a-c). ACPs conditioned medium did not influence cell proliferation compared with the control medium (pristine DMEM). Cell viability was maintained at a high level of (98.8 ± 1.2) % for LO2 and (98.3 ± 1.6) % for Caco-2 after being cultured in ACPs conditioned medium for 72 h, which was comparable to the control. The cell viability of LO2 and Caco-2 were also comparable after 24 or 48 h exposure of cells to ACPs or not (Additional file 1: Fig. S6). Notably, we aimed to explore the cell viability and cell proliferation of LO2 and Caco-2 under ACPs or Control, instead of comparing the quantity of both LO2 and Caco-2 under ACPs. Two weeks after implantation, blood was collected for analysis. The blood analyses of inflammatory cells including white blood cell (WBC), neutrophil (NEU), monocyte (MON), and lymphocyte (LYMPH) were comparable among the healthy group, fibrin group, and ACPs group, indicating the good biocompatibility of ACPs (Fig. 4a).

We also investigated the degree of inflammation and the morphological changes of ACPs after they were implanted in dorsal subcutaneous rat models for 2 weeks. We observed the morphological changes of ACPs with time in the histological images, demonstrating the degradation of ACPs in vivo (Fig. 4b-d). The histological assessment indicated that the inflammatory reaction triggered by ACPs was mild, and was comparable to that caused by fibrin, a commercial product of tissue adhesive (Fig. 4d, e). We also observed the morphological changes of ACPs with time in the histological images, demonstrating the degradation of ACPs in vivo (Fig. 4b-d). We collected the blood from healthy rats, as well as the rats that were implanted with fibrin or ACPs for 2 weeks. The blood analyses of rats were comparable among the 3 groups without marked signs of inflammation and systemic toxicity (Additional file 1: Fig. S10).

### Bridging tissues with arbitrary surface topographies

**Fig. 5:**
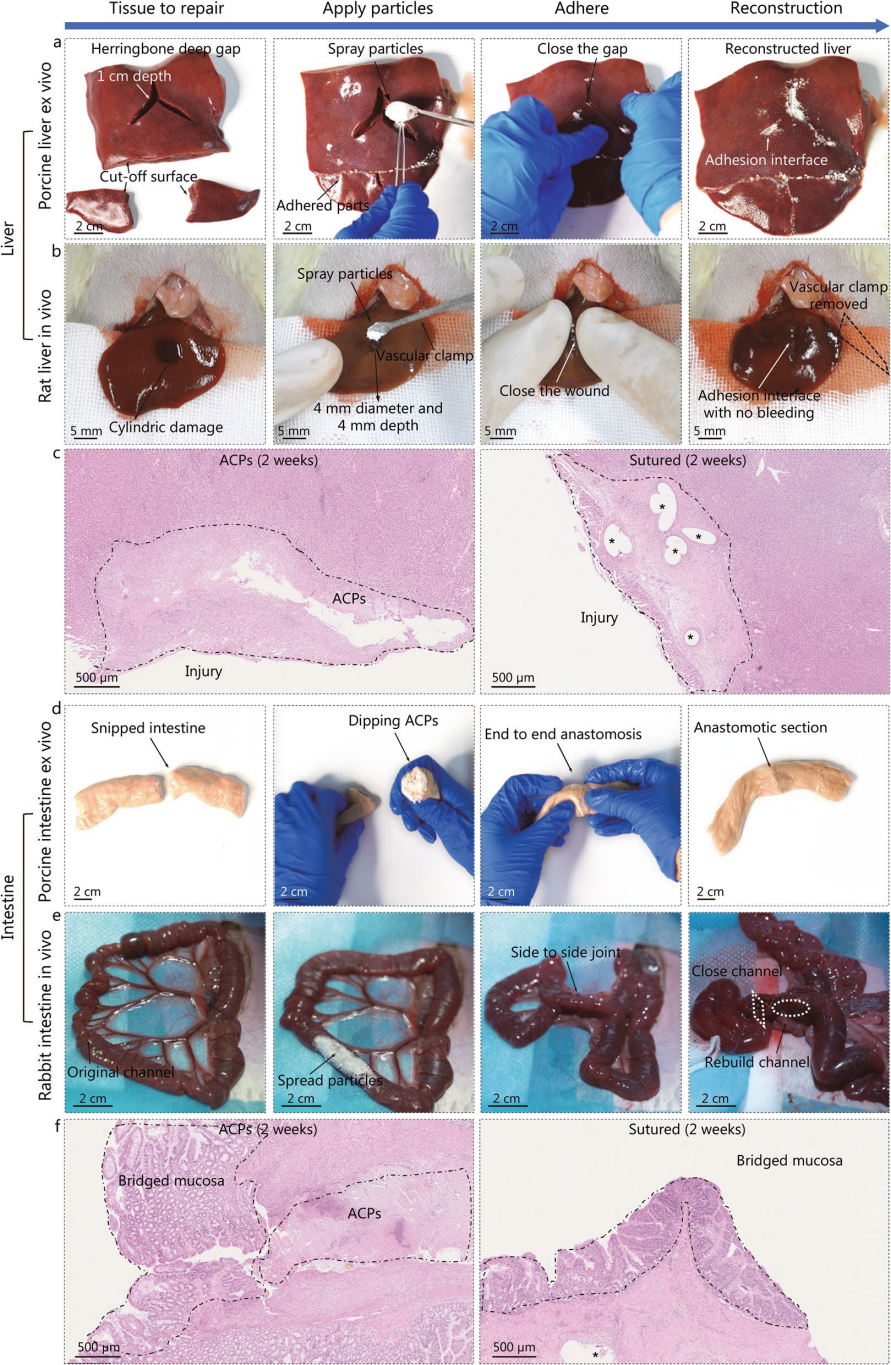
Applications of ACPs in vivo and ex vivo. **a** A porcine liver was cut into three pieces with a severe wound in the shape of deep herringbone grooves ex vivo. After the tissue interfaces were bridged using ACPs, the liver was reconstructed. **b** A severe cylindric wound was introduced to a rat liver in vivo. After bridging the inner surface of the damage with ACPs, the wound was closed without bleeding.** c** Representative histological images of the rat liver, after being damaged and then bridged by ACPs **(left)** or repaired by suturing **(right)** for two weeks. **d** Side-to-side anastomosis of a porcine large intestine using ACPs ex vivo. **e** Side-to-side anastomosis of a rabbit small intestine using ACPs in vivo. **f** Representative histological images of the rabbit small intestine, after they went through side-to-side anastomosis using ACPs (left) or suturing (right) for two weeks. ACPs adhesive cryogel particles

ACPs are adaptable to any surface topography. We illustrated the possible applicability of bridging confined defects in organs with parenchyma and a discontinuous section in cavernous organs with a hollow interior. Parenchyma organs are typically damaged with severe wounds, which always have irregular surface topographies. To demonstrate this, a collection of porcine heart, liver, and kidneys were used as ex vivo models, and a rat liver as the in vivo model (Fig. 5a, b, Additional file 1: Fig. S14a, b). For the ex vivo models, the tissues were cut using a scalpel to cause wounds in the shape of deep herringbone grooves (over 10 mm in depth). The liver was separated into 3 pieces for consequent reconstruction. After removing the remaining blood, ACPs were dusted in the deep wound. The reconstructions of separated tissues were completed after holding the wounds for 10 s. The separated liver was also reconstructed by dipping the interfaces in ACPs and assembling the pieces. The reconstructed tissue interfaces resisted peeling owing to the bridging by ACPs (Additional file 2). As liver rupture is the most common parenchyma organ trauma, requiring liver repair in the emergency setting. The application for tissue-loss defects of ACPs was verified in vivo rat model. Cylindrical holes (4 mm in diameter and 7 mm in depth) were dug as wounds on the liver of the rat model. The wound size is huge considering the diameter of the rat livers (ranging from 20 to 30 mm). After occluding the blood flow using hemostatic forceps and wiping off residual blood, the deep wound was closed with ACPs. No leakage was observed at the site of the repaired wound after the liver was resupplied with blood by opening the forceps (Additional file 3). Five independent in vivo experiments were conducted, and the rat model expressed normal behavior for more than 2 weeks after the surgeries. Histological images acquired two weeks after the operation showed that liver cells grew across the interface with comparable inflammation (Additional file 1: Fig. S10a, while ACPs partly degraded (Fig. 5c). The blood analyses of liver models were comparable among the 3 groups without marked signs of inflammation and systemic toxicity (Additional file 1: Fig. S8).

Cavernous organs are typically damaged by discontinuous cross-sections. This was demonstrated in the ex vivo models of the porcine stomach (Additional file 1: Fig. S14c, Additional file 4) and large intestine (Fig. 5d, Additional file 5), as well as in the in vivo model of the rabbit small intestine (Fig. 5e, Additional file 1: Fig. S14d, Additional file 6, Additional file 7). In the ex vivo models, the stomach was pierced leaving a herringbone incision and the large intestine was snipped into annular sections. After applying ACPs to the external surface of the stomach and around the incision, the surfaces with ACPs were folded and pinched together. Subsequently, the incision was sealed without leakage even when the stomach was filled with water. ACPs were applied to the external surface of the large intestine by turning the intestine inward and dipping. The reconnected large intestine, with a diameter of 30 mm, resisted a pull-off force of 4.7 N before rupture (Additional file 1: Fig. S15). Compared to the burst pressure of 3.7 kPa from the tightly stitched intestine that leaked air through the pinhole, ACPs bridged the intestine with a resistance pressure of 7.8 kPa before the adhesion at the interface failed (Additional file 1: Fig. S16, Additional file 8). As intestinal rupture is the most common cavernous organ trauma, requiring intestinal anastomosis for the reconstruction of digestive tracts. In the in vivo rabbit model, intestinal anastomosis with ACPs was performed (Fig. 5e). During the surgery, we used sterile gauze to separate the surgical area (Additional file 1: Fig. S17a), avoiding the risk of abdominal adhesions. Meanwhile, we also checked the bowel patency after intestinal anastomosis considering the residual powder intraluminally (Additional file 1: Fig. S17b). On week 2 postoperatively, we did not observe any abdominal adhesions or intestinal obstruction in ACPs group (Additional file 1: Fig. S17c). The model rabbits expressed normal behavior 2 weeks after the operation. The intestine mucosa was reconstructed and bridged within 2 weeks with comparable inflammation (Fig. 5f and Additional file 1: Fig. S10b). In the operation, bridging the intestines by ACPs takes less than 30 s, which is remarkably faster than suturing, which takes more than 10 min.

## Discussion

Various tissue adhesives, ranging from cytotoxic to biocompatible and from weak adhesion to strong adhesion, have been developed. Most adhesives are only suitable for wound sealing and hemostatic functions, which is insufficient for treating serious injuries such as organ ruptures and digestive system tears. These injuries necessitate bridging distinct tissues with strong tissue-tissue adhesions, which presents extra difficulties and renders the majority of adhesives useless. Particularly, adhesions are formed between tissues, which necessitates that the adhesives be biodegradable; otherwise, they will get embedded in the healing tissues. Additionally, they must be discontinuous to maintain mass transfer and tissue growth across the interface. Moreover, separated tissues typically have irregular surface topographies, and the ability of adhesives to conform to confined and irregular interfaces is significant. This research aimed to develop tissue adhesives in the form of ACPs. ACPs can be obtained from a fast, mild, and economic preparation. It is also capable of reaching confined and irregular tissue defects for application. In addition to meeting the biocompatibility and adhesion requirements of tissue adhesives, they also meet the criterion of bridging distinct tissues. ACPs are biodegradable and permit discontinuous tissue interfaces. In addition, the application of ACPs is straightforward, which is advantageous for a variety of surgical procedures because it reduces the time required for tissue rebuilding. We compared the performance of ACPs with tissue adhesives in recent reports, including hydrogel tape [24, 29], paste [37], fibrin gel, UV-curable surgical glue [42] and cyanoacrylate glue, GI patch [43], coacervate-derived hydrogel [39], adhesive powder [36] (Table 1). ACPs have high adhesion energy, rapid adhesion formation, excellent biocompatibility, biodegradability, and morphological adaptability. Although it requires carefully spread to avoid unexpected contact of ACPs with irrelevant tissue during the application of ACPs, it still has good enough maneuverability in clinical practice.

**Table 1:** Comparison of adhesion performance between ACPs and various existing tissue adhesives

Tissue adhesives	Adhesion energy (J/m^2^)	Application time for adhesion formation (s)	Biocompatibility	Biodegradability	Morphological adaptability	Maneuverability
ACPs	671 ± 50	< 10	Excellent	Excellent	Excellent	Good
Hydrogel tape [24]	~ 710	1–5	Excellent	Excellent	Good	Excellent
Paste [37]	~ 330	5–15	Good	Fair	Excellent	Good
Nanoparticle solution [21]	25 ± 5	~ 30	-	-	Fair	Fair
Fibrin gel [24]	< 20	> 60	Excellent	Excellent	Good	Good
UV-curable surgical glue [42]	-	5–30	Excellent	-	Fair	Good
Cyanoacrylate glue [24]	< 100	10–60	Fair	Fair	Good	Good
Electro-Ox hydrogel tape [29]	1268	10–21,600	Excellent	Excellent	Good	Excellent
GI patch [43]	560	< 10	Excellent	Excellent	Good	Excellent
Adhesive powder [36]	-	~ 10	Excellent	Fair	Excellent	Good
Coacervate-derived hydrogel [39]	-	~ 600	Excellent	Fair	Excellent	Good

-no report, ACPs adhesive cryogel particles

In this study, the adhesion and biological performance of ACPs were characterized. The adhesion energies to various tissues are considerable. It is a notable difference in adhesion energy among different tissue substrates. This tissue-dependent adhesion also exists in recent reports of strong tissue adhesion based on tough hydrogels. For example, a tough alginate-polyacrylamide hydrogel showed different adhesion energy in various tissues (about 900 J/m^2^ for skin, about 900 J/m^2^ for cartilage, about 600 J/m^2^ for heart, about 600 J/m^2^ for artery and about 200 J/m^2^ for liver) [44]. Dry hydrogel tape also showed this trend (more than 710 J/m^2^ for skin, 580 J/m^2^ for small intestine, 450 J/m^2^ for stomach, 570 J/m^2^ for muscle, 340 J/m^2^ for heart, and 190 J/m^2^ for liver) [24]. Bridging polymer chitosan-based adhesion also exhibits tissue related adhesion energy (about 10 J/m^2^ for liver, about 25 J/m^2^ for heart, about 35 J/m^2^ for artery, and about 90 J/m^2^ for skin) [27]. The tissue-dependent interfacial toughness is a complex problem coupled with biochemistry and mechanics, which remains largely unexplored. However, there is a general hypothesis that the tissue-dependent adhesion performance is related to the mechanical properties of the tissues and their interfacial interactions with the hydrogels [45].

ACPs also exhibited excellent biocompatibility comparable to commercial fibrin gels. The biodegradability was validated by the enzyme solution, tissue adhesion test, and in vivo experiments. Due to the biodegradability and biocompatibility, ACPs have potential drug delivery and wound management application. ACPs are quite suitable and have advantages in medical settings where the tissues are severely injured or dissected having various confined and irregular defects. We have demonstrated the application of ACPs in mending some severely injured organs and intestinal anastomosis. Although we did not define the specific disease model, the surgical procedure of side-to-side intestinal anastomosis is suitable for diseases requiring segmental resection for intestinal reconstruction such as intestinal tumors, inflammatory bowel disease, and intestinal perforation [46–48]. Other unstudied organs and even cartilaginous tissue can also be repaired by ACPs. ACPs may be utilized in further surgical procedures, including lumbar discectomy and ligament restoration, as well as gastrointestinal and pancreatic anastomosis. In percutaneous injuries, when the muscle and the skin are involved, and the tissue surfaces are regular, adhesive tapes and some commercial hemostatic products could be better choices. Injuries of the intestine are common during wartime, which demands urgent diagnostic procedures and emergency treatment [49]. ACPs offer a favorable solution for battlefield rescue and pre-hospital care of intestinal injuries, due to their simple storage, high portability, and ease of manufacturing. Furthermore, frontline medics are able to close defects of the intestine in a few minutes using ACPs with minimal training.

The study has some limitations. First, although we noted that the adhesion energy varies for different tissues, the current study merely measures the adhesion energy to several porcine organs, additional adhesion performances to more tissues should be measured for more applications. Second, for existing ACPs, the pace of degradation is not modifiable; however, there is an alternative degradable crosslinker, and the rate of degradation should be adjusted by swapping crosslinkers. Third, all animals are followed for only two weeks after ACP-related procedures; additional long-term investigations are required for comprehensive biological evaluations. Fourth, excess ACPs spread outside the defect area by incautious handing may lead to abdominal adhesion and even intestinal obstruction in surgical practice. ACPs require more precise dosage control methods, and electrostatic spraying is a potentially effective method.

## Conclusions

In conclusion, ACPs exhibit superior mechanical properties, biocompatibility with tissues, and adhesion to arbitrary surface topographies. ACPs provide a possible method for bridging tissues in future therapeutic procedures. The fast approach of repairing confined and irregular tissue defects by ACPs may provide a basis for further studies on strategies of battlefield rescue.

## Supplementary Information

**Additional file 1**: **Fig. S1**. Preparation of ACPs. **Fig. S2.** Adhesion energy test of various tissues bridged by ACPs. **Fig. S3.** Scanning electron microscope images of adhesion interfaces by ACPs. **Fig. S4.** M Transmission FTIR spectrum of ACPs. **Fig. S5**. Quantification of residual monomer in ACPs by HPLC **Fig. S6**. Confocal microscopy images of the live/dead assay of LO2 and Caco-2. **Fig. S7**. The blood analysis result of the rats 2 weeks after dorsal subcutaneous implantation. **Fig. S8**. The blood analysis results of rats after liver surgeries for 2 weeks. **Fig. S9**. The blood analysis results of rabbits after intestine anastomosis for 2 weeks. **Fig. S10**. Histological assessment for the rabbit small intestine and the rat liver in vivo. **Fig. S11**. Formation and degradation of ACPs’ polymer network. **Fig. S12**. Mechanical property of the chitosan crosslinked PAAc hydrogel. **Fig. S13**. Mechanical properties of hydrogels by aggregating ACPs in water. **Fig. S14**. Ex vivo demonstration of ACPs’ applications. **Fig. S15**. Tensile test of the reconstructed porcine large intestine using suturing and ACPs. **Fig. S16**. Bursting pressure of reconstructed porcine large intestine by suturing and ACPs. **Fig. S17**. Prevention of abdominal adhesions for side-to-side intestinal anastomosis with ACPs for in vivo rabbit model.

**Additional file 2**: Repair of the damaged porcine liver by ACPs ex vivo.

**Additional file 3**: ACPs bridge a bleeding rat liver in vivo.

**Additional file 4**: Repair of a pierced porcine stomach by ACPs ex vivo.

**Additional file 5**: Repair of a snipped porcine intestine by ACPs ex vivo.

**Additional file 6**: Side-to-side intestinal anastomosis by ACPs in vivo.

**Additional file 7**: End-to-end intestinal anastomosis by ACPs in vivo.

**Additional file 8**: Burst pressure of porcine intestine bridged by ACPs and suturing.

## Data Availability

The datasets used during the current study are available from the corresponding author on reasonable request.

## Abbreviations

AAc: Acrylic acid
AAm: Acrylamide
ACPs: Adhesive cryogel particles
Caco-2: Human intestinal epithelial cells
DPBS: Dulbecco’s phosphate-buffered saline
DMEM: Dulbecco’s modified Eagle medium
EDC: 1-Ethyl-3-(3-dimethylaminopropyl) carbodiimide
FTIR: Fourier transform infrared spectroscope
GT: Granulation tissue
HPF: High-power field
HPLC: High-performance liquid chromatography
LO2: Human normal liver cells
LYMPH: Lymphocyte
MBAA: N, N’-Methylenebisacrylamide
MON: Monocyte
NEU: Neutrophil
NHS: N-hydroxysuccinimide
SEM: Scanning electron microscopy
SM: Skeletal muscle
UV: Ultraviolet
WBC: White blood cell
