# Supplementary material for: Adhesive cryogel particles for bridging confined and irregular tissue defects
Source: Mil Med Res. 2023 Mar 23;10:15. doi: 10.1186/s40779-023-00451-1 (PMC10035260; doi:10.1186/s40779-023-00451-1)
Supplement: Supplementary file 1 — Additional file 1: Fig. S1. Preparation of ACPs. Fig. S2. Adhesion energy test of various tissues bridged by ACPs. Fig. S3. Scanning electron microscope images of adhesion interfaces by ACPs. Fig. S4. M Transmission FTIR spectrum of ACPs. Fig. S5. Quantification of residual monomer in ACPs by HPLC Fig. S6. Confocal microscopy images of the live/dead assay of LO2 and Caco-2. Fig. S7. The blood analysis result of the rats 2 weeks after dorsal subcutaneous implantation. Fig. S8. The blood analysis results of rats after liver surgeries for 2 weeks. Fig. S9. The blood analysis results of rabbits after intestine anastomosis for 2 weeks. Fig. S10. Histological assessment for the rabbit small intestine and the rat liver in vivo. Fig. S11. Formation and degradation of ACPs’ polymer network. Fig. S12. Mechanical property of the chitosan crosslinked PAAc hydrogel. Fig. S13. Mechanical properties of hydrogels by aggregating ACPs in water. Fig. S14. Ex vivo demonstration of ACPs’ applications. Fig. S15. Tensile test of the reconstructed porcine large intestine using suturing and ACPs. Fig. S16. Bursting pressure of reconstructed porcine large intestine by suturing and ACPs. Fig. S17. Prevention of abdominal adhesions for side-to-side intestinal anastomosis with ACPs for in vivo rabbit model. [file 40779_2023_451_MOESM1_ESM.pdf]

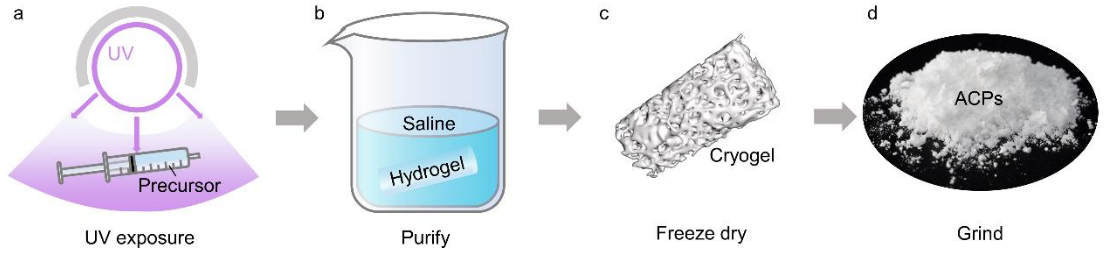

Fig. S1 Preparation of ACPs. **a** The precursor of an adhesive hydrogel is cured under UV exposure. **b** The as-prepared adhesive hydrogel is purified in saline. **c** The purified adhesive hydrogel is freeze-dried into a cryogel. **d** The cryogel is ground into particles with diameters ~10 μm. ACPs adhesive cryogel particles, UV ultraviolet

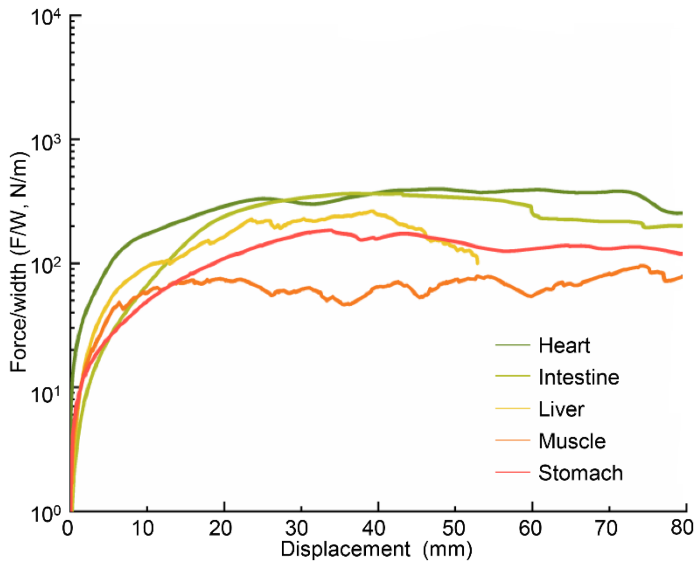

Fig. S2 Adhesion energy test of various tissues bridged by ACPs. Representative force-displacement curves of peeling tissues bridged by ACPs. ACPs adhesive cryogel particles

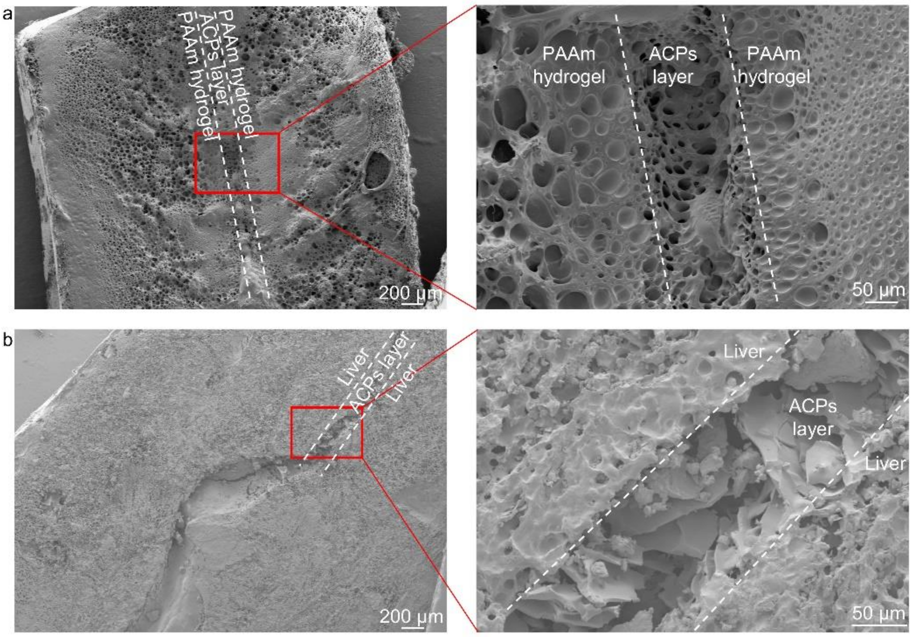

Fig. S3 Scanning electron microscope images of adhesion interfaces by ACPs. **a** Two pieces of PAAm hydrogels are bridged together by ACPs. The polymer network of ACPs is connected with that of the PAAm hydrogels. **b** Two pieces of porcine liver are bridged together by ACPs. The polymer network of ACPs is connected to the liver. The polymer network of ACPs is different when they are applied between the PAAm hydrogels and the liver, this may attribute to the degradation of ACPs by tissue fluid from the liver. ACPs adhesive cryogel particles, PAAm polyacrylamide.

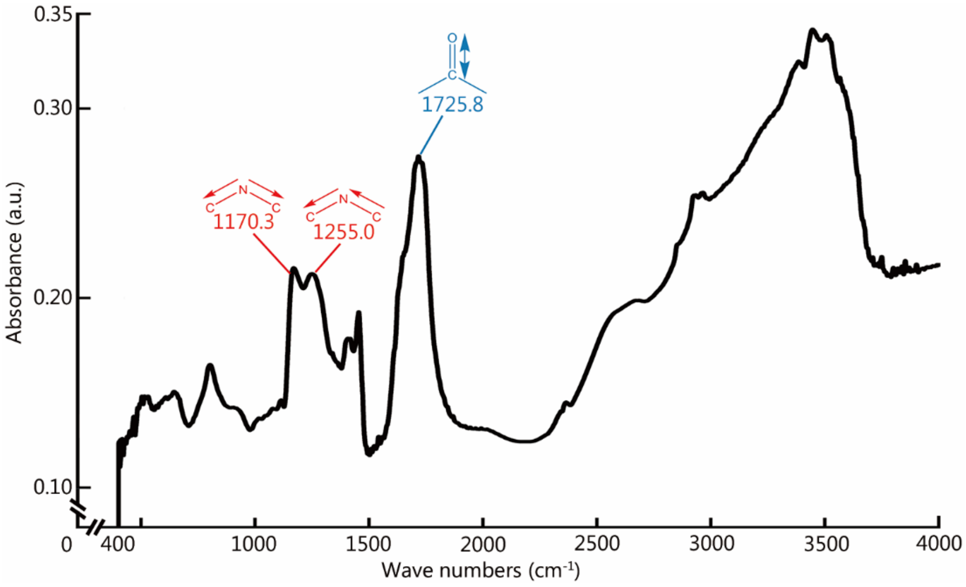

Fig. S4 Transmission FTIR spectrum of ACPs. The carboxylic acid C=O stretch at 1716 cm^{-1} is associated with PAAc in the ACPs. The symmetric C–N–C stretch at 1168 cm^{-1} and asymmetric C–N–C stretch at 1243 cm^{-1} are associated with condensation between the carboxyl groups on polyacrylic acid condense and the amino groups on chitosan chains, indicating the formation of the biodegradable crosslinker. FTIR Fourier transform infrared spectroscope, ACPs adhesive cryogel particles

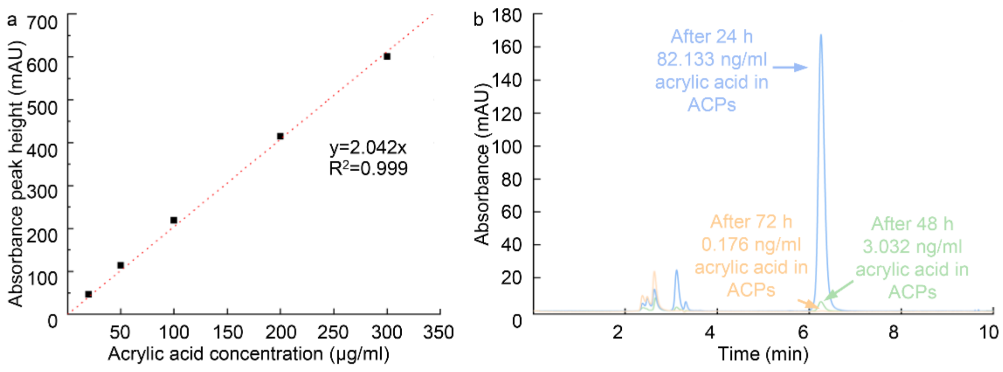

Fig. S5 Quantification of residual monomer in ACPs by HPLC. **a** Standard calibration curve of acrylic acid for HPLC. **b** Results of HPLC characterization of the ACPs' extraction solution on day 1, day 2, and day 3. After 3 days of purification, the ACPs have a very low concentration of residual acrylic acid monomers: 0.176 ng/ml of the ACPs' extraction solution. ACPs adhesive cryogel particles, HPLC high-performance liquid chromatography

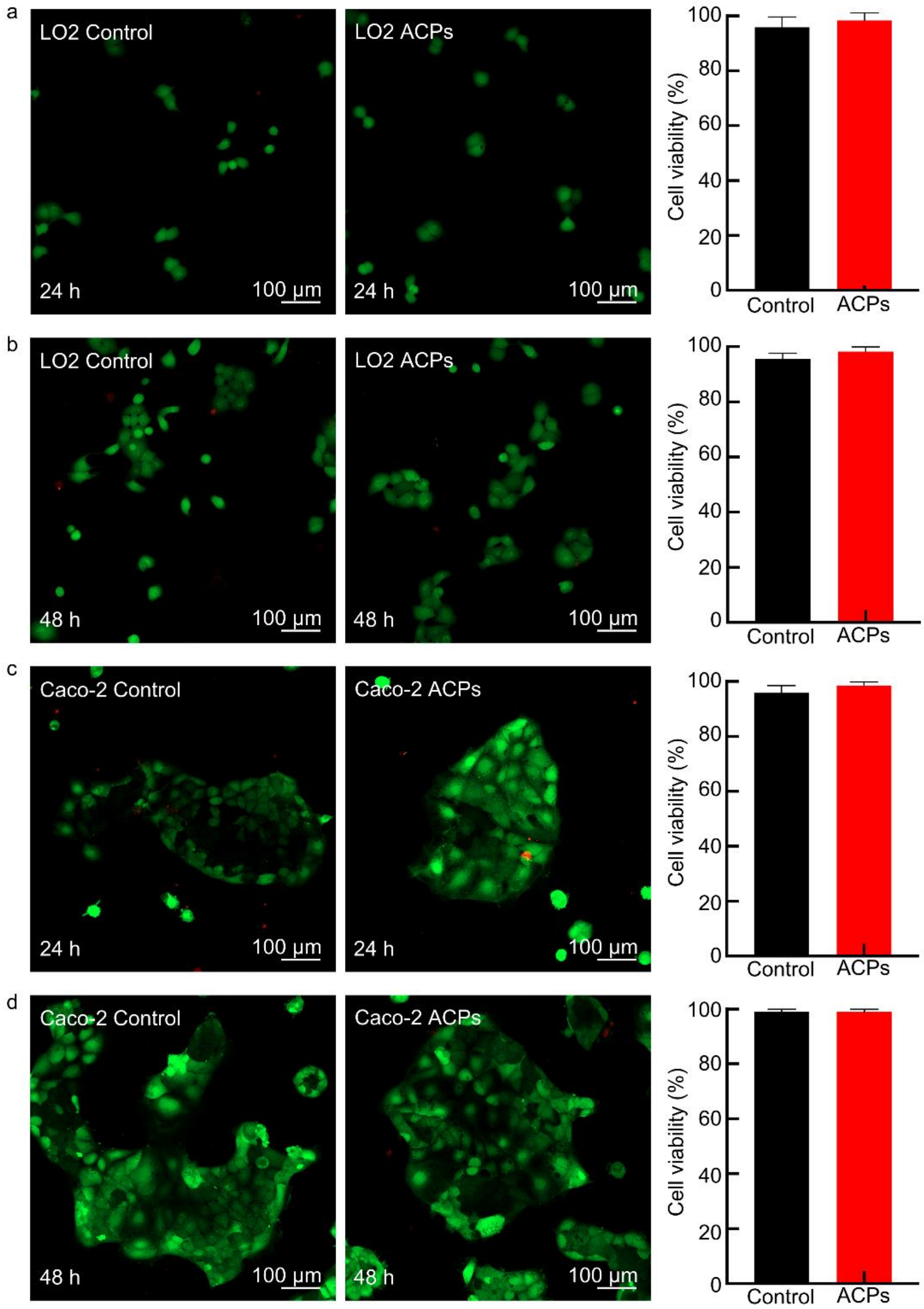

Fig. S6 Confocal microscopy images of the live/dead assay of LO2 and Caco-2. **a**, **b** LO2 cultured in control medium and ACPs-conditioned medium (**right**) and corresponding cell viability (**left**) for 1 day (**a**), and 2 days (**b**). **c**, **d** Caco-2 cultured in control medium and ACPs-conditioned medium (**left**) and corresponding cell viability (**right**) for 1 day (**c**), and 2 days (**d**). ACPs adhesive cryogel particles

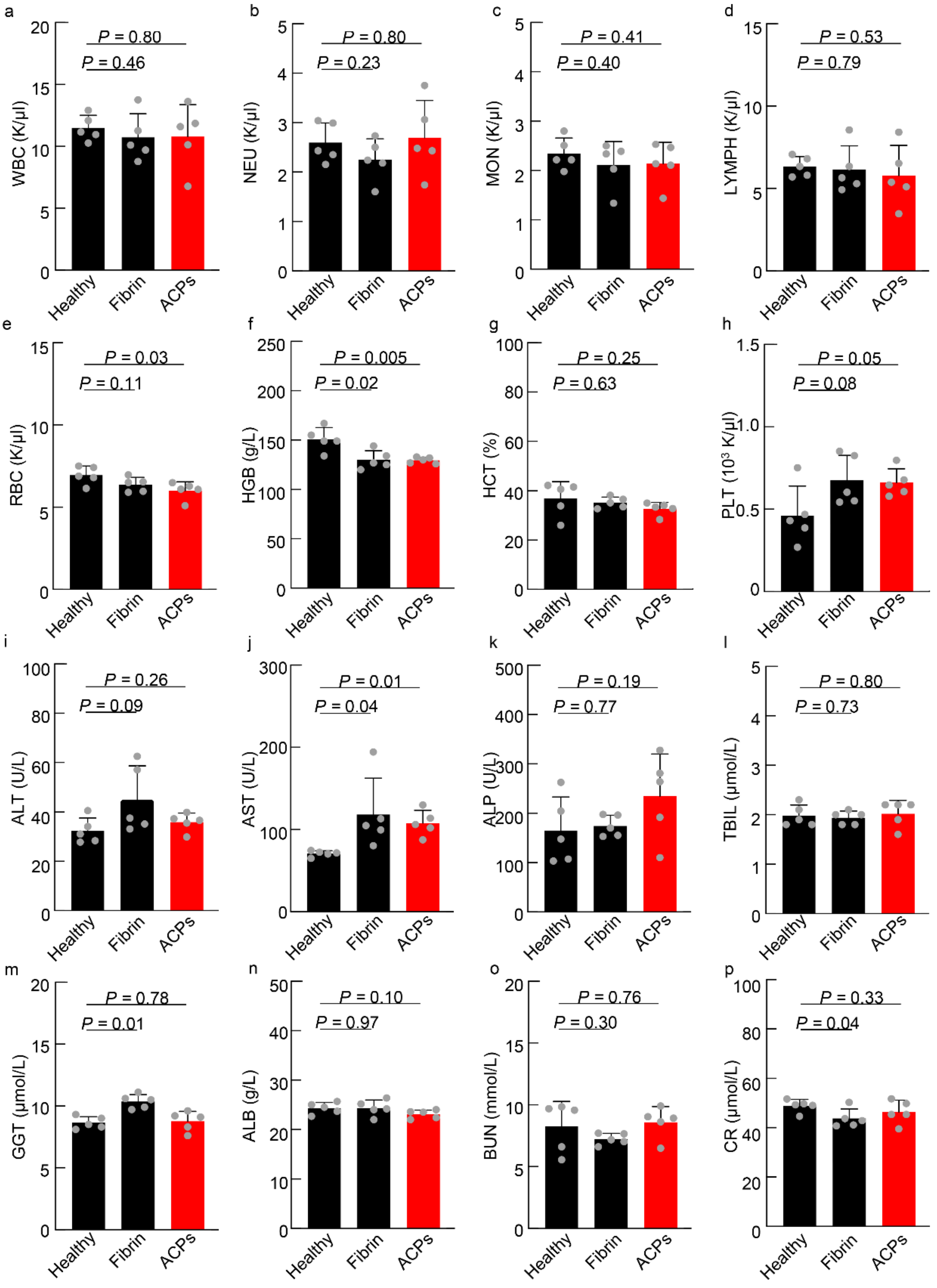

Fig. S7 The blood analysis result of the rats 2 weeks after dorsal subcutaneous implantation. The white blood cell (WBC, **a**), neutrophil (NEU, **b**), monocyte (MON, **c**), lymphocyte (LYMPH, **d**), red blood cell (RBC, **e**), hemoglobin (HGB, **f**), hematocrit (HCT, **g**), platelet (PLT, **h**), alanine Transaminase (ALT, **i**), aspartate Aminotransferase (AST, **j**), alkaline phosphatase (ALP, **k**), total bilirubin (TBIL, **l**), γ-glutamyltransferase

(GGT, **m**), albumin (ALB, **n**), blood urea nitrogen (BUN, **o**), and creatinine (CR, **p**) values are comparable for the rats implanted with ACPs to those that are healthy or implanted with Fibrin gels. ACPs adhesive cryogel particles

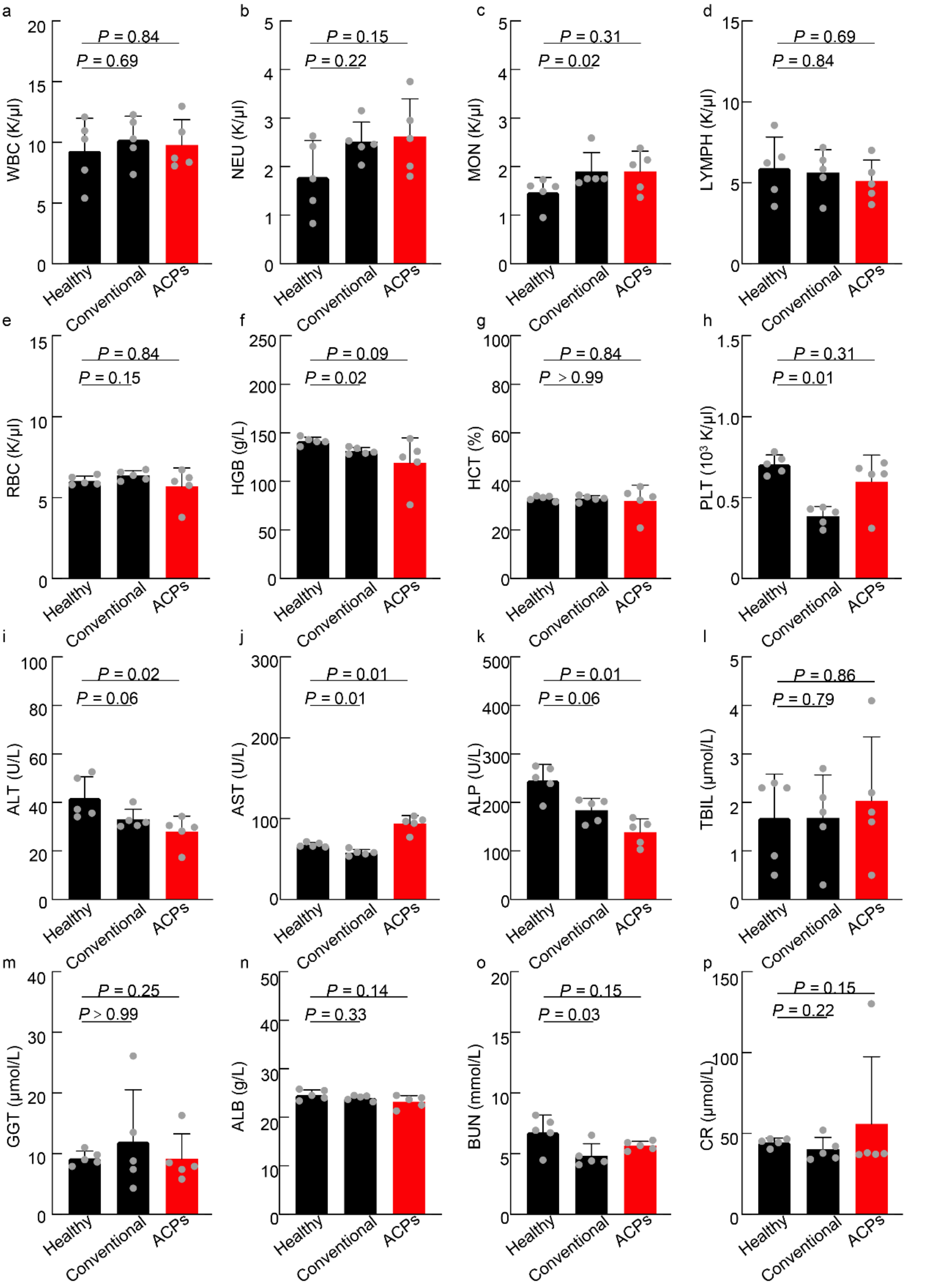

Fig. S8 The blood analysis results of rats after liver surgeries for 2 weeks. The white blood cell (WBC, **a**), neutrophil (NEU, **b**), monocyte (MON, **c**), lymphocyte (LYMPH, **d**), red blood cell (RBC, **e**), hemoglobin (HGB, **f**), hematocrit (HCT, **g**), platelet (PLT, **h**), alanine Transaminase (ALT, **i**), aspartate Aminotransferase (AST, **j**), alkaline

phosphatase (ALP, **k**), total bilirubin (TBIL, **l**), γ-glutamyltransferase (GGT, **m**), albumin (ALB, **n**), blood urea nitrogen (BUN, **o**), and creatinine (CR, **p**) values are comparable for the rats, whose livers are repaired with ACPs, to those that are healthy or repaired by suturing. ACPs adhesive cryogel particles

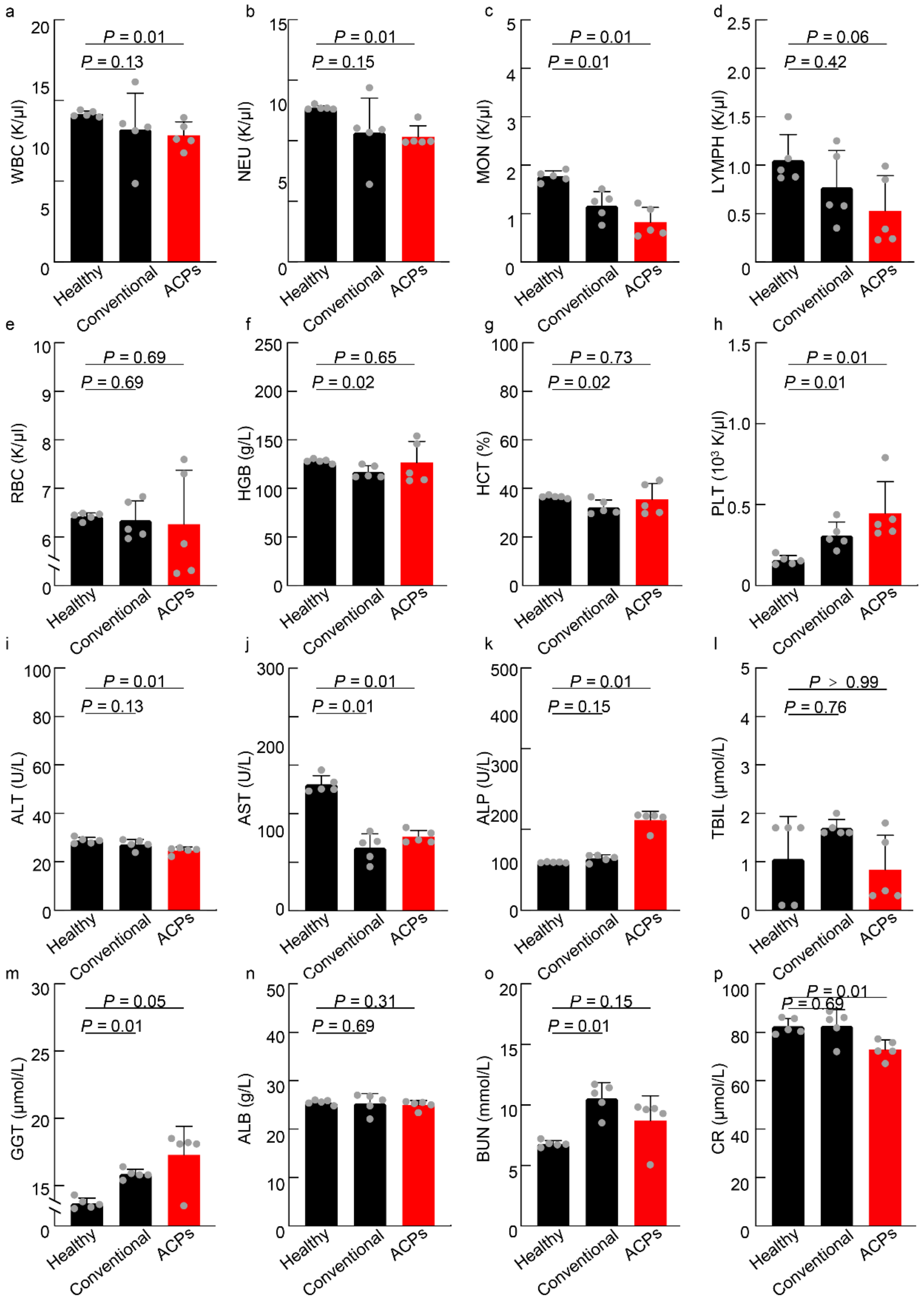

Fig. S9 The blood analysis results of rabbits after intestine anastomosis for 2 weeks. The white blood cell (WBC, **a**), neutrophil (NEU, **b**), monocyte (MON, **c**), lymphocyte (LYMPH, **d**), red blood cell (RBC, **e**), hemoglobin (HGB, **f**), hematocrit (HCT, **g**),

platelet (PLT, **h**), alanine Transaminase (ALT, **i**), aspartate Aminotransferase (AST, **j**), alkaline phosphatase (ALP, **k**), total bilirubin (TBIL, **l**), γ-glutamyltransferase (GGT, **m**), albumin (ALB, **n**), blood urea nitrogen (BUN, **o**), and creatinine (CR, p) values are comparable for the rabbits, who went through intestine anastomosis using ACPs, to those who are healthy or went through intestine anastomosis by suturing. ACPs adhesive cryogel particles

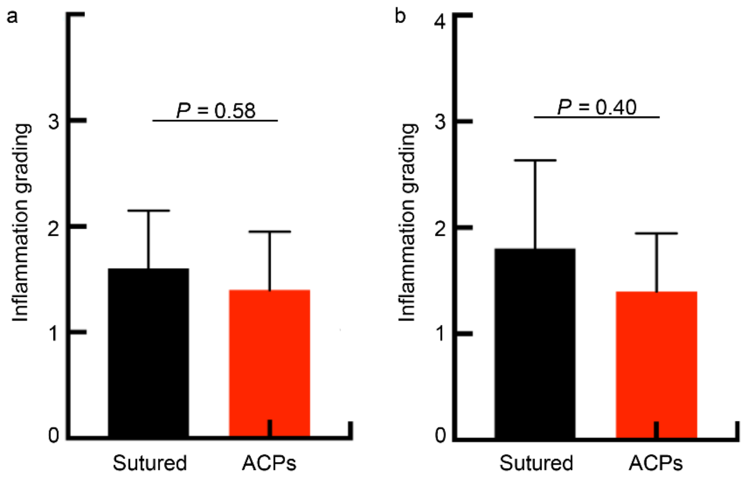

Fig. S10 Histological assessment for the rabbit small intestine and the rat liver in vivo. **a** Inflammation grading for rabbits after intestine anastomosis for 2 weeks. **b** Inflammation grading for rats after liver surgeries for 2 weeks. ACPs adhesive cryogel particles

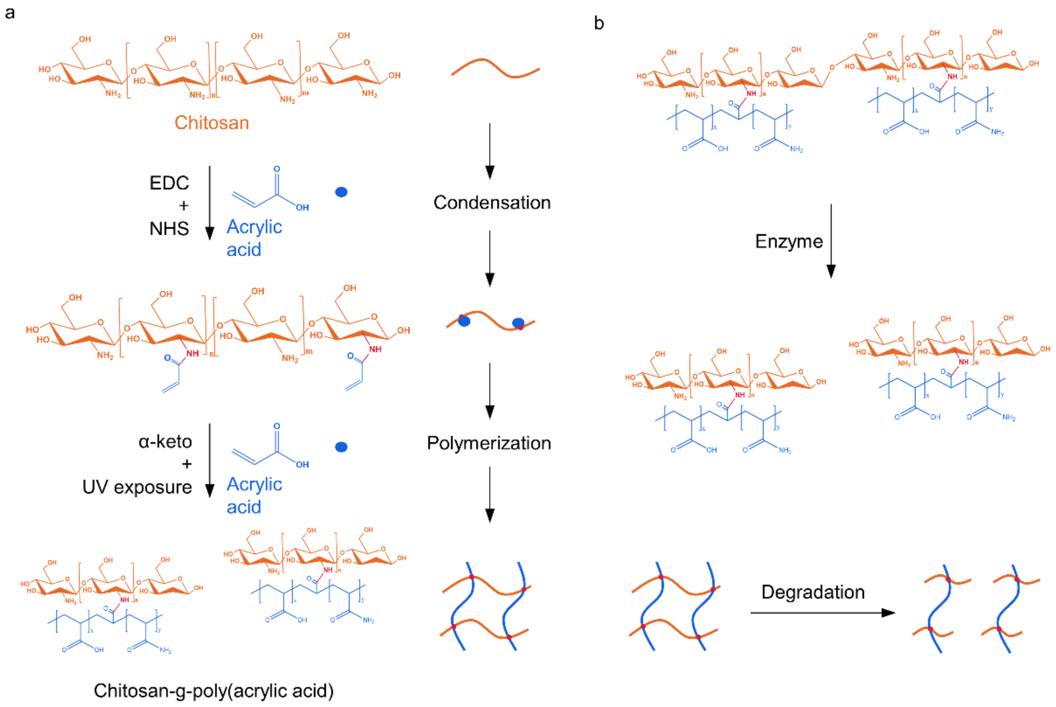

Fig. S11 Formation and degradation of ACPs' polymer network. **a** The carboxyl groups on polyacrylic acid monomers condense with the amino groups on chitosan chains by catalysis of EDC/NHS. The acrylic acid monomers polymerize into long chains by the initiation of α-ketoglutaric acid under UV exposure. **b** A chitosan chain cleaves in presence of enzymes. The polymer network breaks into dissolvable polymer chains. ACPs adhesive cryogel particles, EDC 1-Ethyl-3-(3-dimethylaminopropyl) carbodiimide, NHS N-hydroxysuccinimide UV ultraviolet

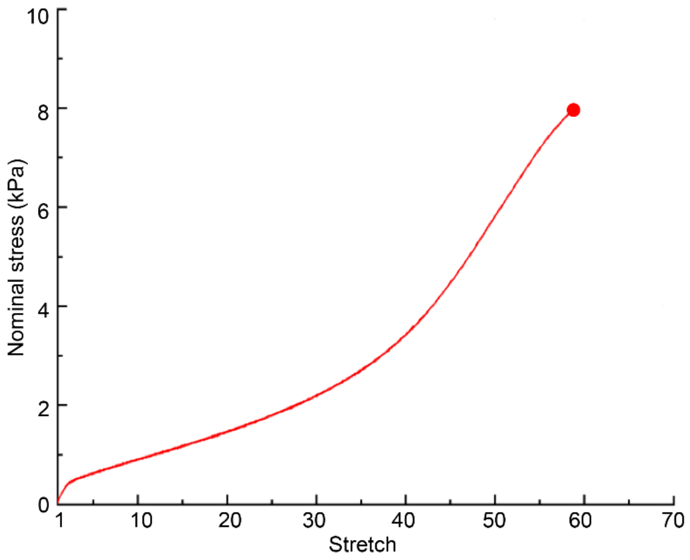

Fig. S12 Mechanical property of the chitosan crosslinked PAAc hydrogel. Representative stress-strain curve of chitosan crosslinked PAAc hydrogel. PAAc polyacrylamide.

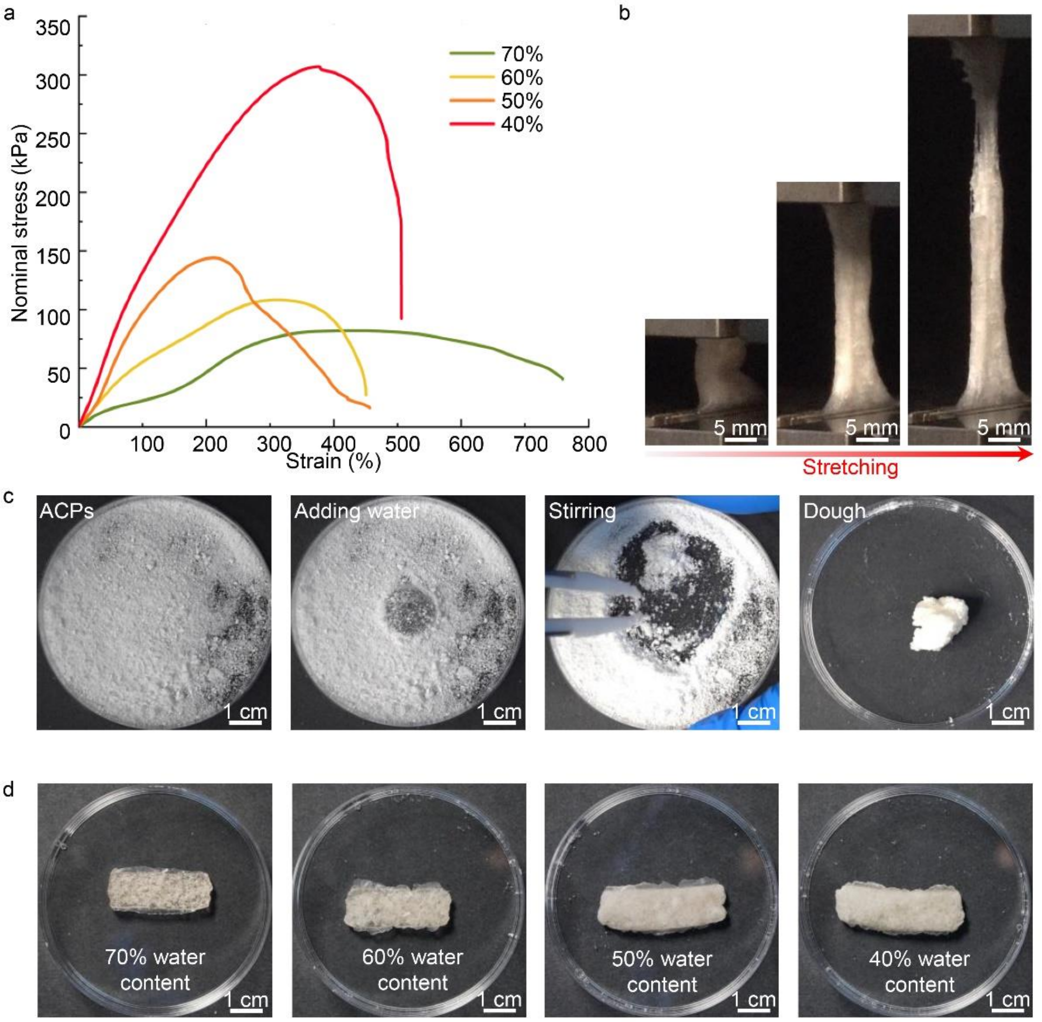

Fig. S13 Mechanical properties of hydrogels by aggregating ACPs in water. **a** Stress-strain curve for ACPs-aggregated hydrogel with water content between 40 % and 70 %. **b** Representative image of tensile test for ACPs-aggregated hydrogel (Sample with 40% water content). **c** Representative images in ACPs aggregating process (Sample with 60% water content). **d** The ACPs-aggregated hydrogel is modeled as tensile test samples (10 mm in width, and 3 mm in thickness). ACPs adhesive cryogel particles

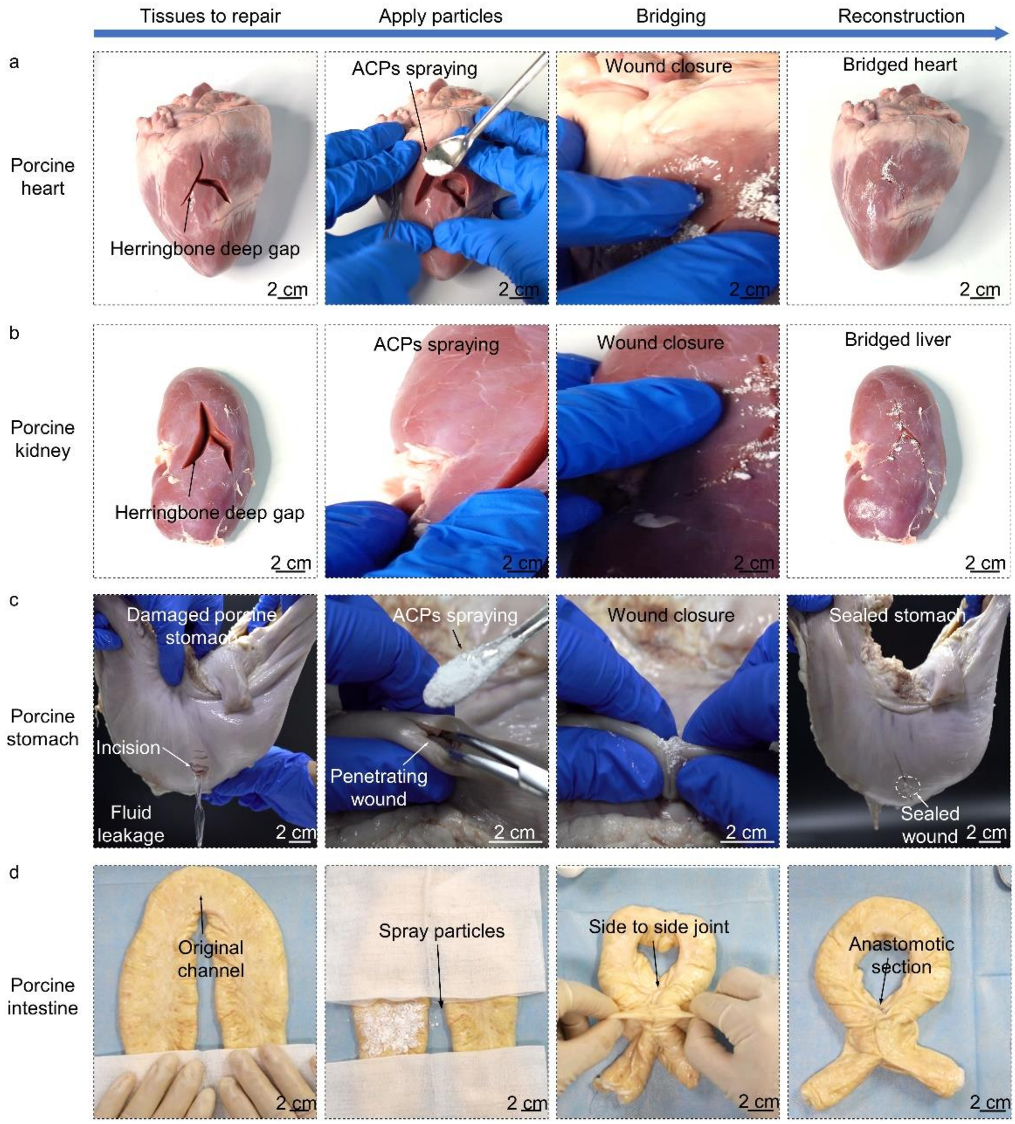

Fig. S14 Ex vivo demonstration of ACPs' applications. A porcine heart **(a)** and a porcine kidney **(b)** were cut, and have wounds in the shape of deep herringbone grooves. After applying ACPs at the tissue interfaces, the wounds are closed for 10 s, and the organs were repaired. **c** The repair of the pierced porcine stomach using ACPs. **d** The end-to-end anastomosis of a porcine large intestine using ACPs. ACPs adhesive cryogel particles

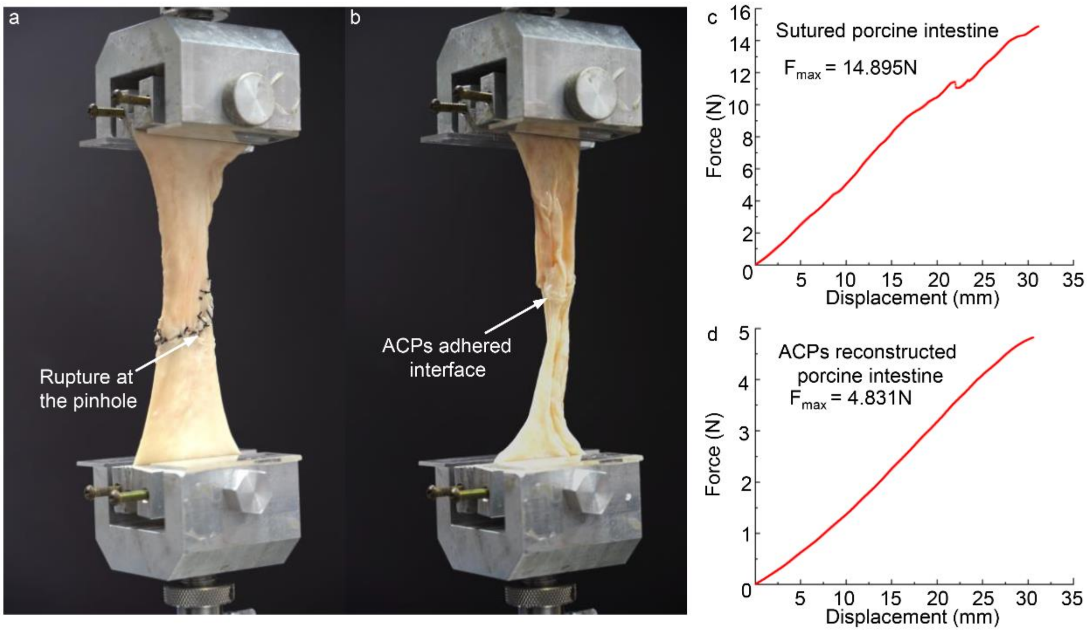

Fig. S15 Tensile test of the reconstructed porcine large intestine using suturing and ACPs. **a** sutured porcine large intestine ruptured from the pinholes. **b** ACPs bridged porcine large intestine ruptured through the adhesive layer. **c**, **d** Force-displacement curve of porcine large intestine reconstructed by suturing (**c**) and ACPs (**d**). ACPs adhesive cryogel particles

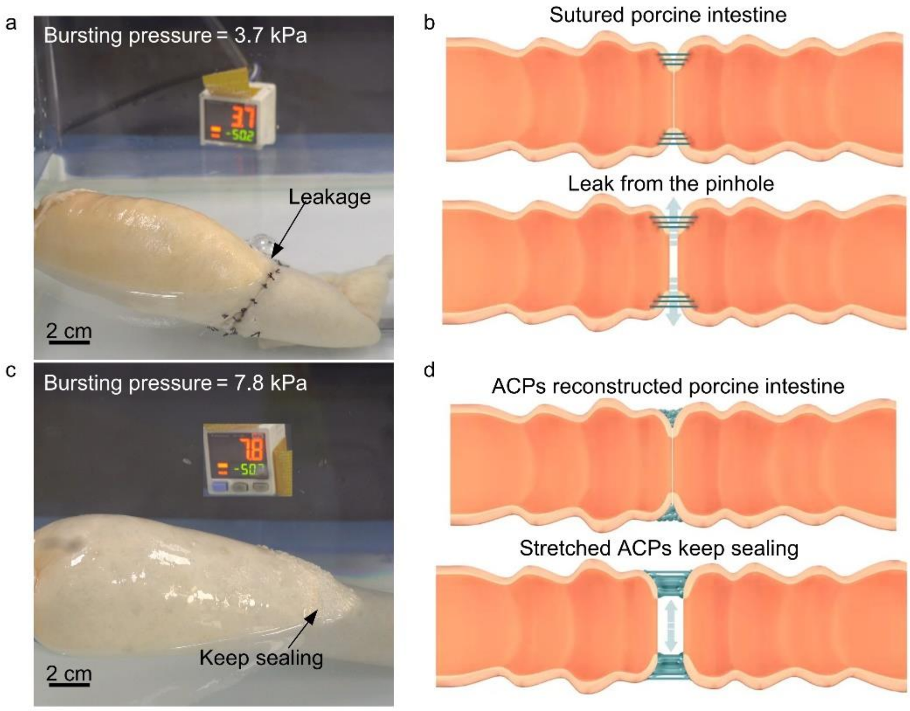

Fig. S16 Bursting pressure of reconstructed porcine large intestine by suturing and ACPs. **a** Leakage occurs at a bursting pressure of 3.7 kPa for sutured intestine. **b** The sutured intestine has rich pinholes that may suffer leakage. **c** Leakage occurs at a bursting pressure of 7.8 kPa for ACPs bridged intestine. **d** The ACPs keep sealing at the interface even when they swell into hydrogels and are stretched. ACPs adhesive cryogel particles

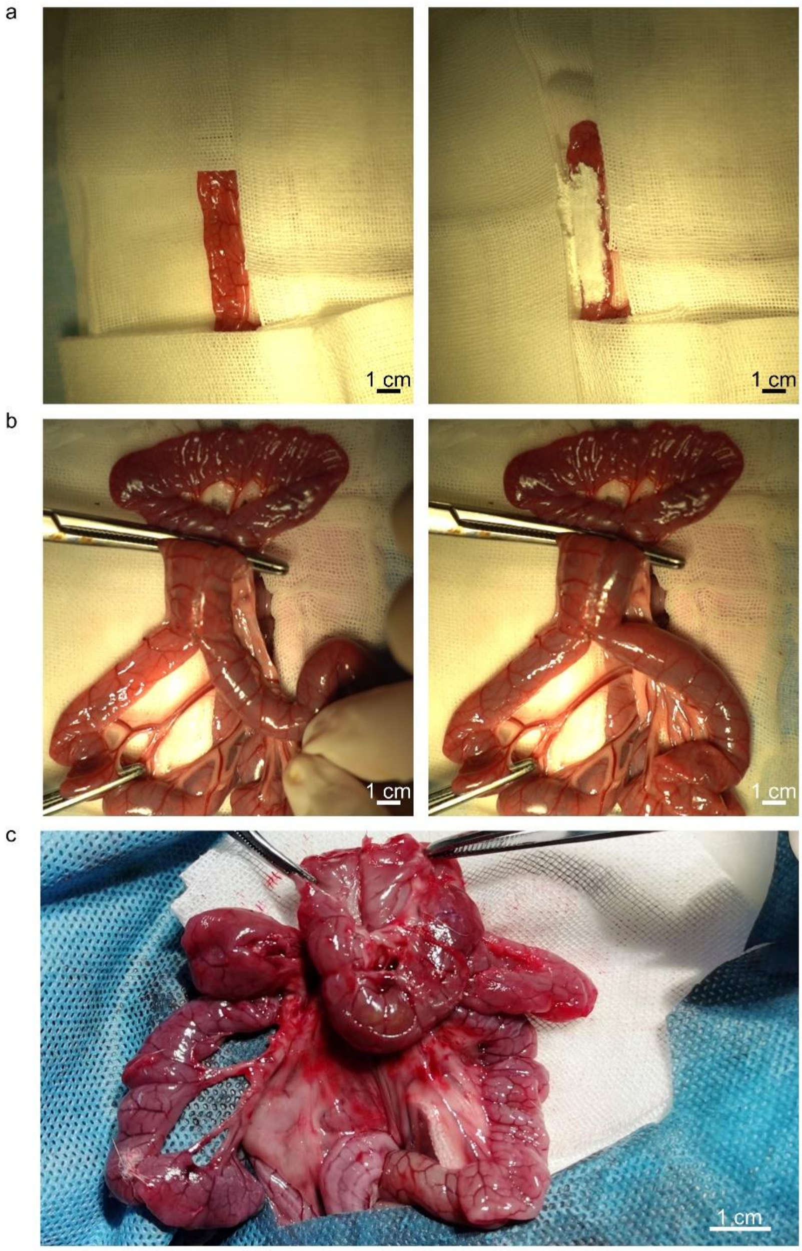

Fig. S17 Prevention of abdominal adhesions for side-to-side intestinal anastomosis with ACPs for in vivo rabbit model. **a** Covering the non-surgical area with sterile gauze. **b** Checking the bowel patency after intestinal anastomosis. **c** No abdominal adhesions or intestinal obstruction happened on week 2 postoperatively. ACPs adhesive cryogel particles
